# Supplementary material for: Phosphoglycerate kinase 1 acts as a cargo adaptor to promote EGFR transport to the lysosome
Source: Nat Commun. 2024 Feb 3;15:1021. doi: 10.1038/s41467-024-45443-4 (PMC10838266; doi:10.1038/s41467-024-45443-4)
Supplement: Supplementary file 1 — Supplementary Information [file 41467_2024_45443_MOESM1_ESM.pdf]

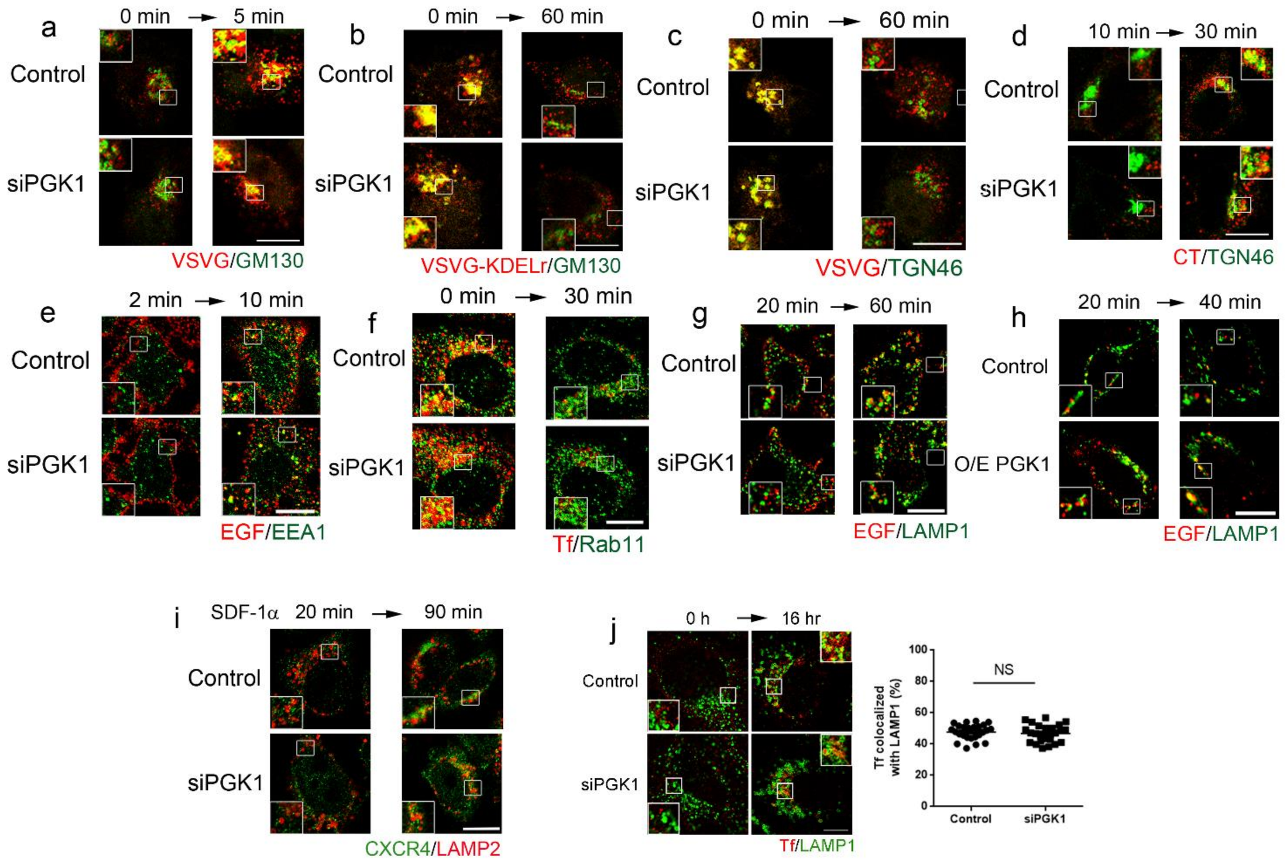

Supplementary Figure 1. Representative images in the screening of intracellular pathways.

a. Representative confocal images of VSVG (red) colocalizing with GM130 (green) at times indicated, bar=10 μm.

b. Representative confocal images of VSVG-KDELr (red) colocalizing with GM130 (green) at times indicated, bar=10 μm.

c. Representative confocal images of VSVG (red) colocalizing with TGN46 (green) at times indicated, bar=10 μm.

d. Representative confocal images of CTxB (red) colocalizing with TGN46 (green) at times indicated, bar=10 μm.

e. Representative confocal images of EGF (red) colocalizing with EEA1 (green) at times indicated, bar=10 μm.

f. Representative confocal images of Tf (red) colocalizing with Rab11 (green) at times indicated, bar=10 μm.

g. Representative confocal images of EGF (red) colocalizing with LAMP1 (green) at times indicated, bar=10 μm.

h. Representative confocal images of EGF (red) colocalizing with LAMP1 (green) at times indicated, bar=10 μm.

i. Representative confocal images of CXCR4 (green) colocalizing with LAMP2 (red) at times indicated, bar=10 μm.

j. Assay for TfR transport to the lysosome. Colocalization of Tf (red) with a lysosomal marker (LAMP1, green) in HeLa cells was performed at times indicated, n=25 cells examined over 3

independent experiments. Representative images are shown on left, bar=10µm. Quantitation of the 16-hour time point from a representative experiment is shown on right. Results are shown as mean +/- s.e.m.; P=0.5123 (NS, not significant), unpaired two-sided Student's t-test.

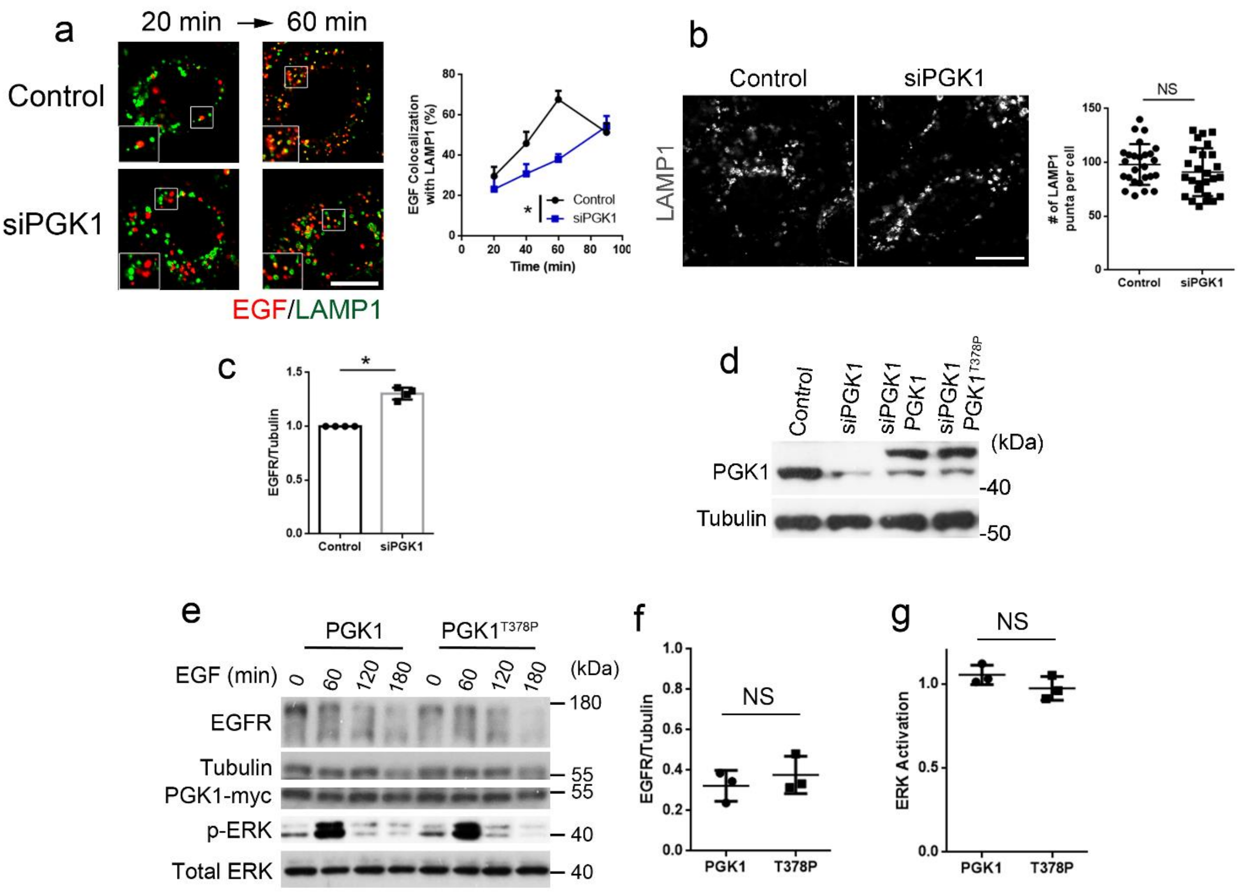

Supplementary Figure 2. Characterizing the role of PGK1 in EGFR transport to the lysosome. Quantitative results are shown as mean +/- s.e.m.; *p<0.05, NS (not significant), unpaired two-sided Student's *t*-test.

a. Assay for endocytic transport of EGFR to the lysosome, examining the effect of siRNA against PGK1. Colocalization of EGF with LAMP1 in A549 cells upon EGF stimulation was performed, n=15 cells examined over 3 independent experiments. Representative images at times indicated are shown on left with EGF in red and LAMP1 in green, bar = 10 µm. Quantitation is shown on right for a representative experiment, with statistics performed for the 60-min time point, P=2.08x10^{-11}.

b. Assessing the number of lysosomes, comparing control versus siPGK1-treated cells, n=26 cells examined over 3 independent experiments. HeLa cells were stained for LAMP1. Representative images are shown on left. Quantitation of LAMP1 punctuates for a representative experiment is shown on right, P=0.1287.

c. EGFR levels at t=0 in Figure 2a, comparing control versus siPGK1(#1), were quantified and then normalized to tubulin levels, n=4, P=0.0016.

d. Immunoblotting of HeLa cell lysate for PGK1 level to assess the efficiency of siRNA against PGK1 and the level of rescue using myc-tagged PGK1 constructs as indicated, n=3. A representative experiment is shown.

e. Time-course analysis of EGFR degradation in HeLa cells upon EGF stimulation for indicated time point, examining the effect of expressing a catalytic dead mutant of PGK1. Cell lysates were immunoblotted for proteins indicated, n=3. A representative result is shown.

f. Quantitation of EGFR level at the 120-min time point for the analysis above. EGFR level was normalized to tubulin level, P=0.1644.

g. Quantitation of ERK activation at the 120-min time point for the analysis above. Phosphorylated ERK level was normalized to total ERK level, P=0.3181.

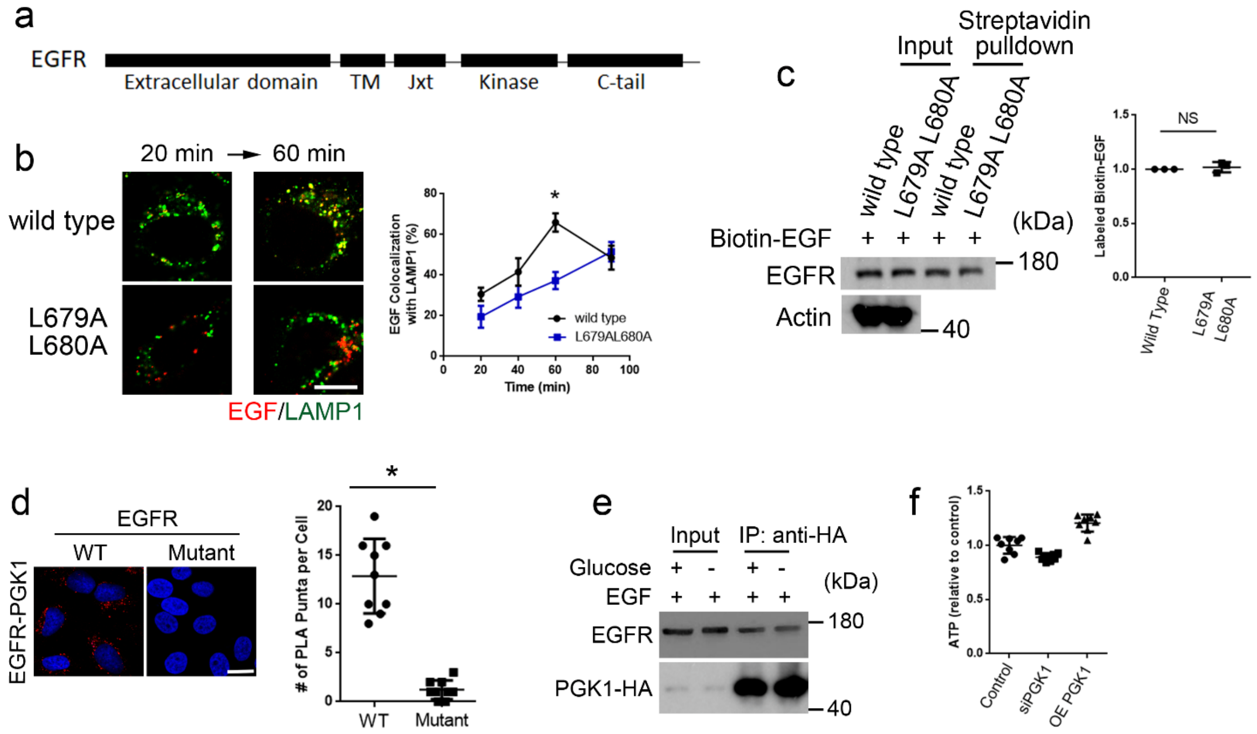

Supplementary Figure 3. Comparing the role of PGK1 in EGFR transport versus metabolism. Quantitative results are shown as mean +/- s.e.m.; *p<0.05, unpaired two-sided Student's *t*-test.

a. Schematic showing the domains of EGFR: extracellular, transmembrane (TM), juxtamembrane region (Jxt), kinase (Kinase), and C-terminal tail (C-tail).

b. Assay for endocytic transport of EGFR to the lysosome, comparing wild-type versus dileucine mutant (L679A/L680A). Colocalization of EGF with LAMP1 upon EGF stimulation was performed at times indicated, n=10 cells examined over 3 independent experiments. Representative images are shown on left, with EGF in red and LAMP1 in green, bar=10 μm. Quantitation of a representative experiment is shown on right, with statistics performed for time = 60 minutes, $P=1.13\times10^{-8}$.

c. Surface EGFR levels were assessed for wild-type versus dileucine mutant. HeLa cells were incubated with biotin-labeled EGF, followed by pulldown using streptavidin beads, n=3. A representative result is shown on left. Quantitation of three experiments is shown on right, with statistics performed for time = 60 minutes, $P=0.552$.

d. PLA analysis examining the effect of mutating the dileucine sorting signal of EGFR on its association with PGK1 in HeLa cells upon EGF stimulation for 1 hour, n=10 cells examined over 3 independent experiments. Puncta tracks the association of PGK1 with EGFR by using primary antibodies directed against endogenous PGK1 and to the myc epitope of the transfected EGFR-myc. Representative images are shown on left, bar= 10 μm. Quantitation is shown on right for a representative experiment, $P=2.27\times10^{-5}$.

e. Examining the effect of glucose starvation on the association of PGK1 with EGFR. HeLa cells were glucose starved for 4 hours and then stimulated with EGF for 1 hour. Cell lysates were then immunoprecipitated for transfected PGK1-HA followed by immunblotting for endogenous EGFR, n=2. A representative result is shown.

f. Quantifying total ATP level upon perturbing PGK1 level in cells. HeLa cells were treated as

indicated, and then cell lysates were measured for total ATP level, n=8.

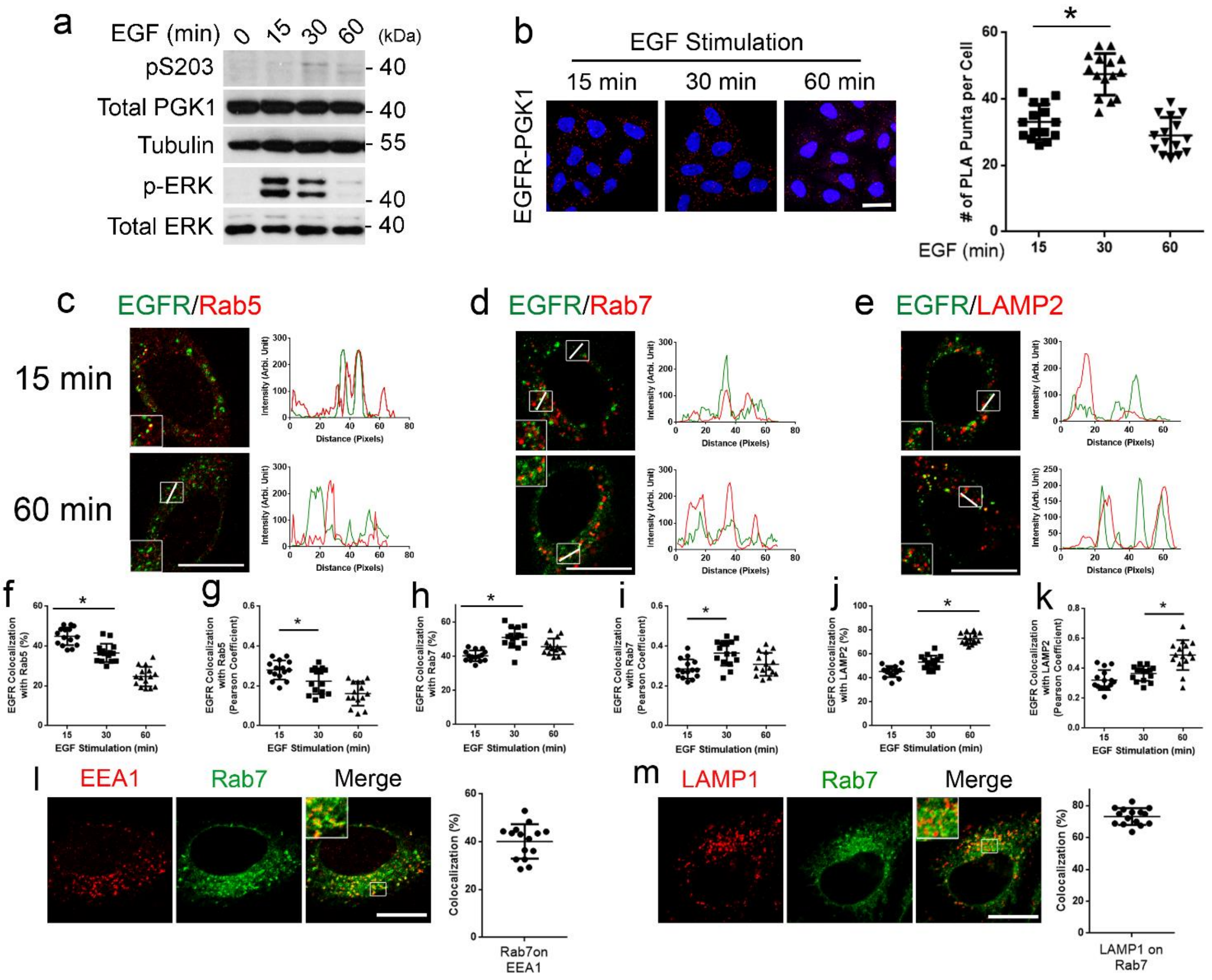

Supplementary Figure 4. Further characterizing the role of PGK1 on EGFR transport. Quantitative results are shown as mean +/- s.e.m.; *p<0.05, unpaired two-sided Student's *t*-test.

a. Time course analysis of S203 phosphorylation on PGK1, n=3.

b. PLA analysis examining the association of PGK1 with EGFR upon EGF stimulation for times indicated, n=15 cells examined over 3 independent experiments, bar = 10 µm. Quantitation is shown, comparting 15- versus 30-min time point, P=1.058x10^{-5}.

c. Time course analysis examining the colocalization of EGFR (green) with Rab5 (red), n=15 cells examined over 3 independent experiments, bar = 10 µm.

d. Time course analysis examining the colocalization of EGFR (green) with Rab7 (red), n=15 cells examined over 3 independent experiments, bar = 10 µm.

e. Time course analysis examining the colocalization of EGFR (green) with LAMP1 (red), n=15 cells examined over 3 independent experiments, bar = 10 µm.

f-g. Quantitation of a representative experiment described above in (c) with result expressed as percent colocalization (f) and Pearson's coefficient (g), and statistical analysis comparing 15-min versus 30-min time point, P=6.87x10^{-4} (percent) 0.00978 (Pearson).

h-i. Quantitation of a representative experiment described above in (d) with result expressed as percent colocalization (h) and Pearson's coefficient (i), and statistical analysis comparing 15-min

versus 30-min time point, P=2.59×10^{-5} (percent) and 0.00451 (Pearson).

j-k. Quantitation of a representative experiment described above in (e) with result expressed as percent colocalization (j) and Pearson's coefficient (k), and statistical analysis comparing 30-min versus 60-min time point, P=1.25×10^{-8} (percent) and 0.0008 (Pearson).

l. Colocalization of EEA1 (red) with Rab7 (green), n=15 cells examined over 3 independent experiments, bar = 10 μm. Quantitation from a representative experiment is shown on right.

m. Colocalization of LAMP1(red) with Rab7 (green), n=15 cells examined over 3 independent experiments, bar = 10 μm. Quantitation from a representative experiment is shown on right.

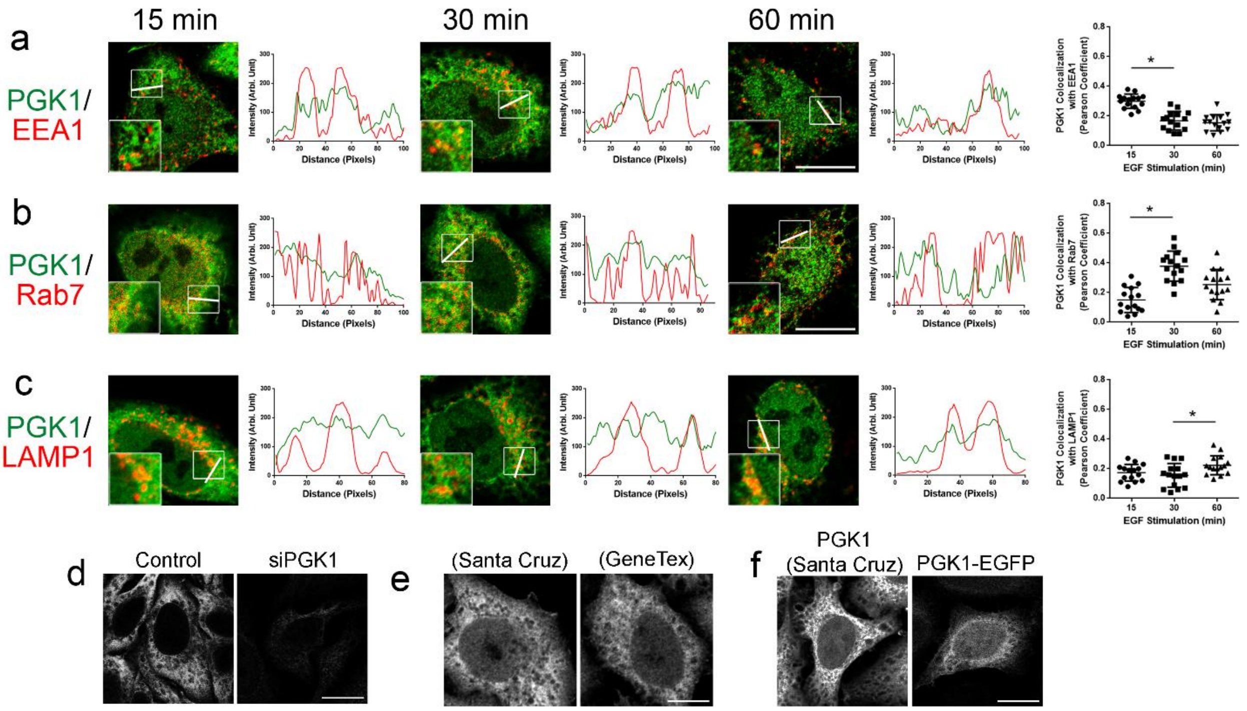

Supplementary Figure 5. Characterizing the distributions PGK1 and endosomal markers. Quantitative results are shown as mean +/- s.e.m.; *p<0.05, unpaired two-sided Student's *t*-test.

a. Time course analysis examining the colocalization of PGK1 with EEA1 (an early endosome marker) in HeLa cells, n=15 cells examined over 3 independent experiments. Antibodies against endogenous proteins are used. Left shows representative images of PGK1 (green) colocalizing with EEA1 (red) upon EGF stimulation at times indicated, bar = 10 μm. Right shows quantitation of a representative experiment with result expressed as Pearson's coefficient, and statistical analysis comparing 15-min versus 30-min time point, P=4.01×10^{-5}.

b. Time course analysis examining the colocalization of PGK1 with Rab7 in HeLa cells, n=15 cells examined over 3 independent experiments. Antibodies against endogenous proteins are used. Left shows representative images of PGK1 (green) colocalizing with Rab7 (red) upon EGF stimulation at times indicated, bar = 10 μm. Right shows quantitation of a representative experiment with result expressed as Pearson's coefficient, and statistical analysis comparing 15-min versus 30-min time point, P=1.47×10^{-6}.

c. Time course analysis examining the colocalization of PGK1 with LAMP1, n=15 cells examined over 3 independent experiments. Antibodies against endogenous proteins are used. Left shows representative images of PGK1 (green) colocalizing with LAMP1 (red) upon EGF stimulation at times indicated, bar = 10 μm. Right shows quantitation of a representative experiment with result expressed as Pearson's coefficient, and statistical analysis comparing 30-min versus 60-min time point, P=0.01645.

d. Comparing antibody staining for PGK1 in control versus si-PGK1 treated HeLa cells, n=3. Representative images are shown, bar = 10 μm.

e. Comparing PGK1 staining in HeLa cells using two different antibodies against PGK1, n=3. Representative images are shown, bar = 10 μm.

f. Comparing the distribution of PGK1 in HeLa cells using antibody against PGK1 versus the expression of GFP-tagged PGK1. Representative images are shown, bar = 10 μm.

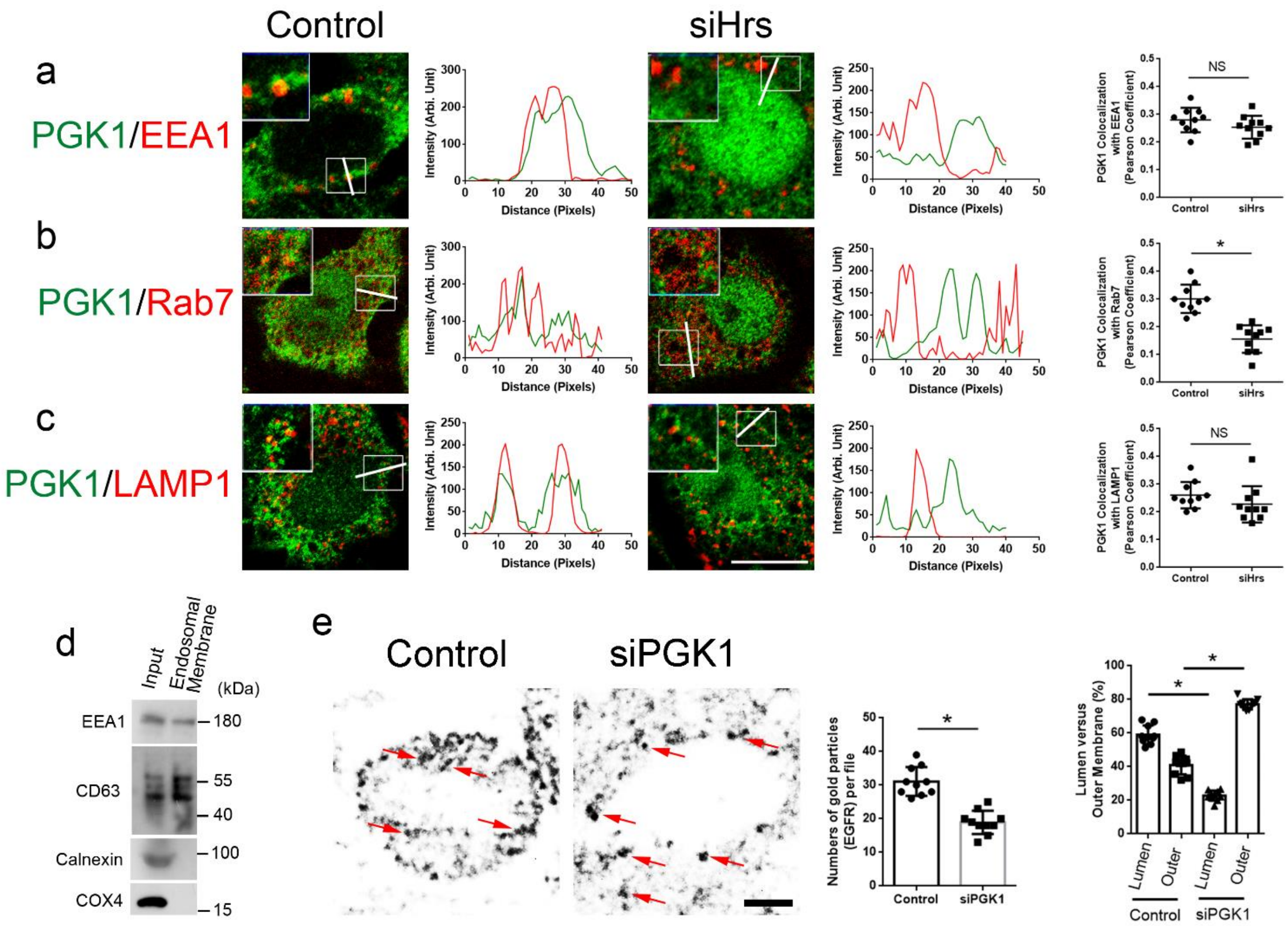

Supplementary Figure 6. Characterizing the effects of siRNA against Hrs and PGK1. Quantitative results are shown as mean +/- s.e.m.; *p<0.05, unpaired two-sided Student's *t*-test.

a. Colocalization of PGK1 with EEA1 in HeLa cells upon EGF stimulation was assessed by confocal microscopy, n=10 cells examined over 3 independent experiments. Representative images are shown on left with PGK1 in green and EEA1 in red, bar = 10 μm. Quantitation from a representative experiment is shown on right, with result expressed as Pearson's coefficient, *P*=0.232.

b. Colocalization of PGK1 with Rab7 in HeLa cells upon EGF stimulation was assessed by confocal microscopy, n=10 cells examined over 3 independent experiments. Representative images are shown on left with PGK1 in green and Rab7 in red, bar = 10 μm. Quantitation from a representative experiment is shown on right, with result expressed as Pearson's coefficient, $P=4.37\times10^{-6}$.

c. Colocalization of PGK1 with LAMP1 upon EGF stimulation was assessed by confocal microscopy, n=10 cells examined over 3 independent experiments. Representative images are shown on left with PGK1 in green and LAMP1 in red, bar = 10 μm. Quantitation from a representative experiment is shown on right, with result expressed as Pearson's coefficient, *P*=0.313.

d. Characterizing the endosomal membrane fraction. The membrane fraction was immunoblotted various organelle markers, EEA1 (early endosome), CD63 (late endosome), calnexin (ER), COX4 (mitochondria).

e. Electron microscopy assessing the level of EGFR at the late endosome. HeLa, either untreated or si-PGK1 treated, were stimulated with EGF for 1 hour, and then immunogold labeled for EGFR, n=10 cells examined over 3 independent experiments. Representative images of the late endosome are

shown on left, bar = 200 nm. Arrowheads indicate gold particles. Quantitation was performed in two ways. The left graph compares the total level of EGFR, P=8.04×10^{-6}. The right graph compares the fraction of EGFR in vesicles (lumen) versus that in the limiting membrane (outer), P=1.566×10^{-4} (lumen), P=1.57×10^{-7} (outer).

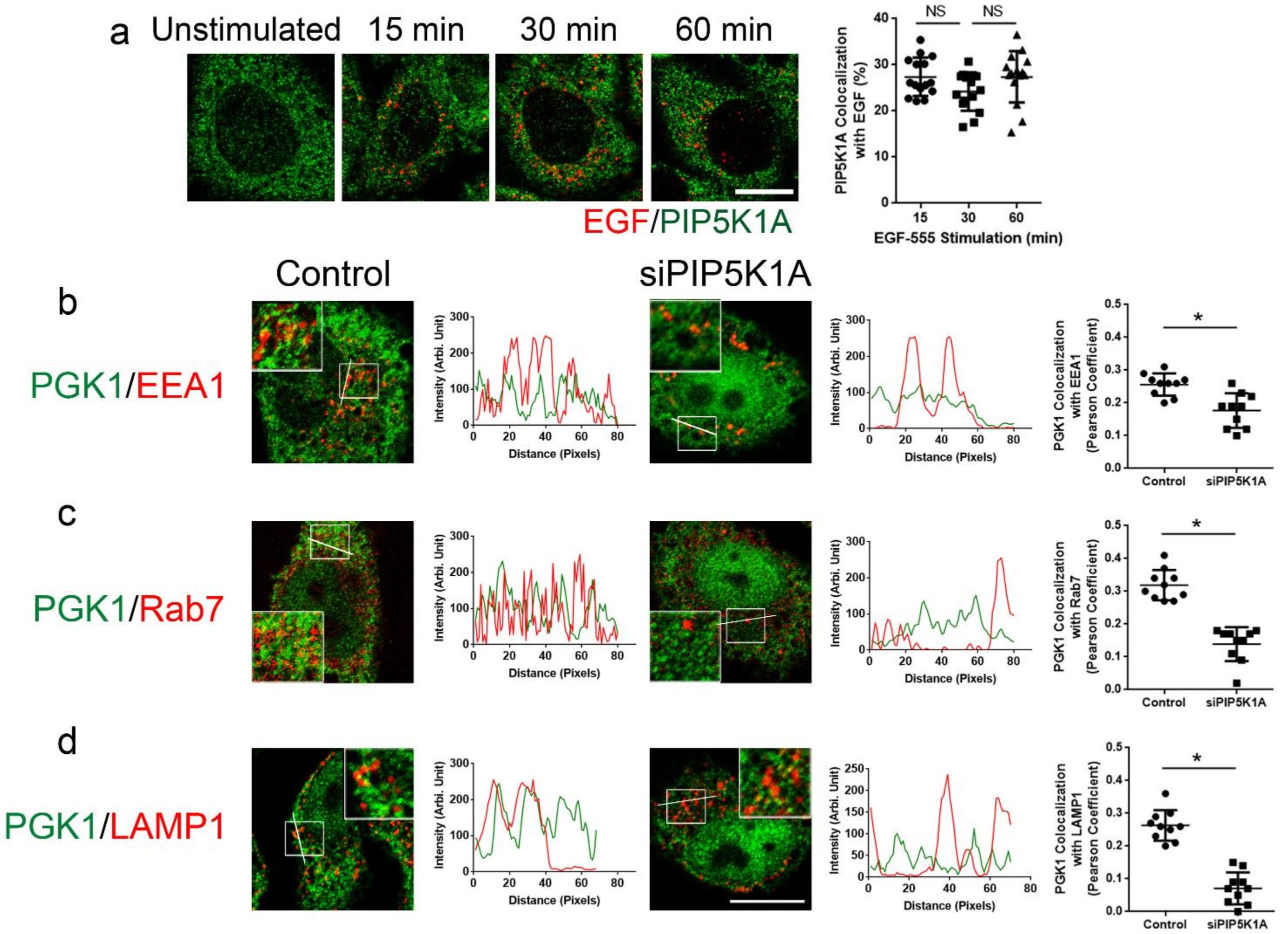

Supplementary Figure 7. Characterizing the effects of siRNA against PIP5K1A.

Quantitative results are shown as mean +/- s.e.m.; $*p<0.05$, NS (not significant), unpaired two-sided Student's *t*-test.

a. Time course analysis examining the colocalization of PGK1 with EGFR upon EGF stimulation by confocal microscopy, n=15 cells examined over 3 independent experiments. Representative images of EGF (red) colocalizing with PIP5K1A (green) at times indicated are shown on left, bar = 10 μm. Quantitation of a representative experiment is shown on right. Statistics was performed comparing colocalizations at 15 minutes versus at 30 minutes, P=0.0522, and at 30 minutes versus at 60 minutes, P=0.0873.

b. Colocalization of PGK1 with EEA1 in HeLa cells upon EGF stimulation was assessed by confocal microscopy, comparing control versus si-PIP5K1A, n=10 cells examined over 3 independent experiments. Representative images are shown on left with PGK1 in green and EEA1 in red, bar = 10 μm. Quantitation from a representative experiment is shown on right, with result expressed as Pearson's coefficient, *P*=0.00327.

c. Colocalization of PGK1 with Rab7 in HeLa cells upon EGF stimulation was assessed by confocal microscopy, comparing control versus si-PIP5K1A, n=10 cells examined over 3 independent experiments. Representative images are shown on left with PGK1 in green and Rab7 in red, bar = 10 μm. Quantitation from a representative experiment is shown on right, with result expressed as Pearson's coefficient, $P=1.952\times10^{-5}$.

d. Colocalization of PGK1 with LAMP1 upon EGF stimulation was assessed by confocal microscopy,

comparing control versus si-PIP5K1A, n=10 cells examined over 3 independent experiments. Representative images are shown on left with PGK1 in green and LAMP1 in red, bar = 10 μm. Quantitation from a representative experiment is shown on right, with result expressed as Pearson's coefficient, P=8.88×10^{-6}.

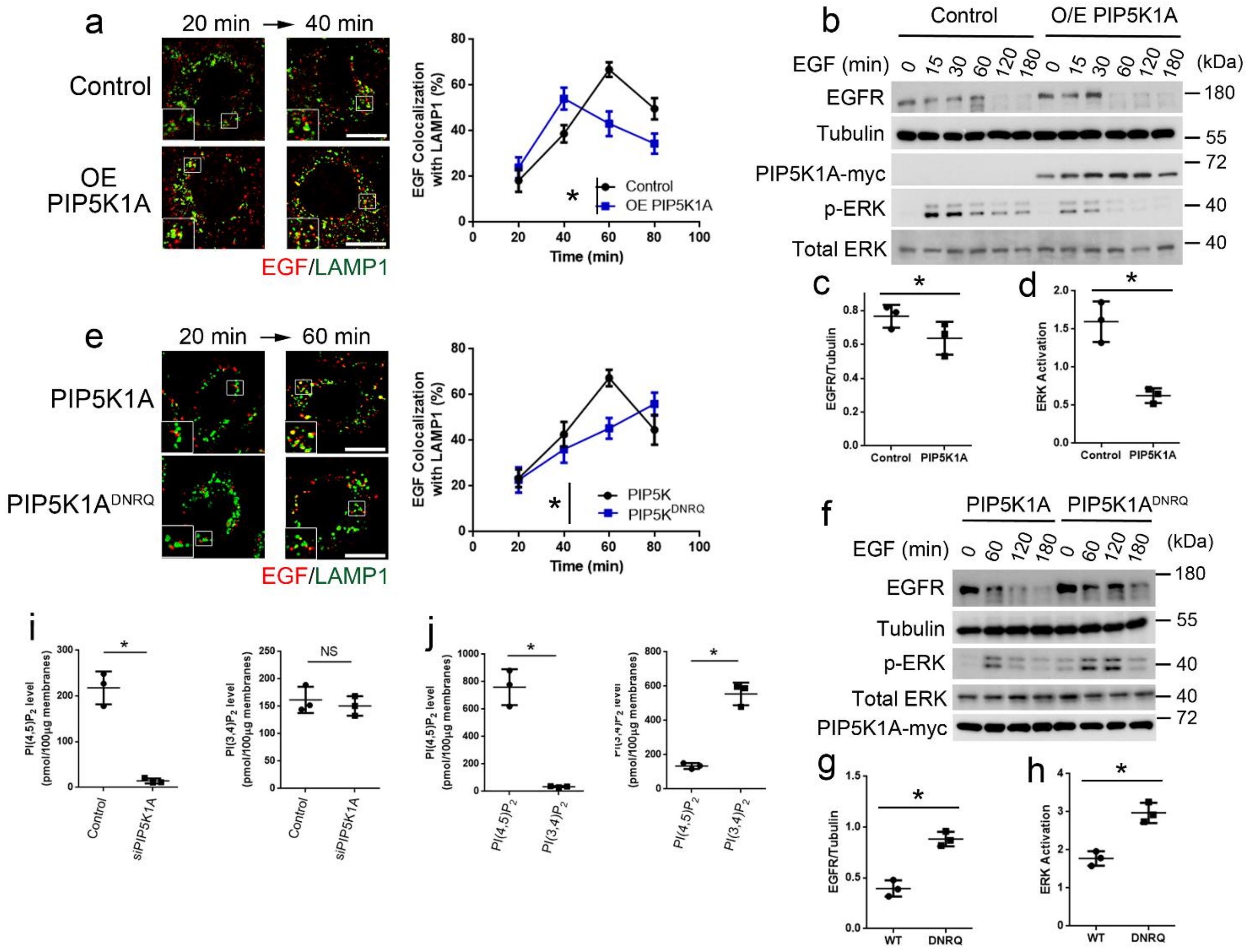

Supplementary Figure 8. Further characterizing the role of PIP5K1A.

Quantitative results are shown as mean +/- s.e.m.; *p<0.05, unpaired two-sided Student's *t*-test.

a. Colocalization of EGF with LAMP1 (green) upon EGF (red) stimulation was performed, n=10 cells examined over 3 independent experiments, bar = 10 µm. Statistics is shown for the 60-min time point, P=2.5x10^{-10}.

b. Time-course analysis of EGFR degradation, examining the effect of PIP5K1A overexpression, n=3.

c. Quantitation of EGFR level at the 60-min time point for the analysis above. EGFR level was normalized to tubulin level, P=0.033.

d. Quantitation of ERK activation at the 60-min time point for the analysis above. Phosphorylated ERK level was normalized to total ERK level, P=0.0049.

e. Colocalization of EGF (red) with LAMP1 (green) upon EGF stimulation was performed, n=10 cells examined over 3 independent experiments, bar = 10 µm. Statistics is shown for the 60-min time point, P=2.24x10^{-10}.

f. Time-course analysis of EGFR degradation, examining the effect of expressing the catalytic dead mutant of PIP5K1A, n=3.

g. Quantitation of EGFR level at the 60-min time point for the analysis above. EGFR level was normalized to tubulin level, P=0.005.

h. Quantitation of ERK activation at the 60-min time point for the analysis above. Phosphorylated ERK level was normalized to total ERK level, P=0.0038.

i. Quantitation of PI(4,5)P2 and PI(3,4)P2 levels on endosomal membrane comparing control versus PIP5K1A-depleted membrane, *P*=0.00839 and 0.3317 for PI(4,5)P2 and PI(3,4)P2 levels, respectively.

j. Quantitation of PI(4,5)P2 and PI(3,4)P2 levels on endosomal membrane comparing the effect of delivering PI(4,5)P2 or PI(3,4)P2 to endosomal membrane by feeding either lipid to cells, *P*=0.01146 and 0.0121 for PI(4,5)P2 and PI(3,4)P2 levels, respectively.
