# Supplementary material for: Phosphoglycerate kinase 1 acts as a cargo adaptor to promote EGFR transport to the lysosome
Source: Nat Commun. 2024 Feb 3;15:1021. doi: 10.1038/s41467-024-45443-4 (PMC10838266; doi:10.1038/s41467-024-45443-4)
Supplement: Supplementary file 3 — Reporting Summary [file 41467_2024_45443_MOESM3_ESM.pdf]

nature portfolio

Corresponding author(s): Jia-Wei Hsu

Last updated by author(s): 01/03/2024

Reporting Summary

Nature Portfolio wishes to improve the reproducibility of the work that we publish. This form provides structure for consistency and transparency in reporting. For further information on Nature Portfolio policies, see our Editorial Policies and the Editorial Policy Checklist.

Statistics

For all statistical analyses, confirm that the following items are present in the figure legend, table legend, main text, or Methods section.

n/a	Confirmed	
☐	☒	The exact sample size (*n*) for each experimental group/condition, given as a discrete number and unit of measurement
☐	☒	A statement on whether measurements were taken from distinct samples or whether the same sample was measured repeatedly
☐	☒	The statistical test(s) used AND whether they are one- or two-sided<br>*Only common tests should be described solely by name; describe more complex techniques in the Methods section.*
☒	☐	A description of all covariates tested
☐	☒	A description of any assumptions or corrections, such as tests of normality and adjustment for multiple comparisons
☐	☒	A full description of the statistical parameters including central tendency (e.g. means) or other basic estimates (e.g. regression coefficient) AND variation (e.g. standard deviation) or associated estimates of uncertainty (e.g. confidence intervals)
☐	☒	For null hypothesis testing, the test statistic (e.g. *F*, *t*, *r*) with confidence intervals, effect sizes, degrees of freedom and *P* value noted<br>*Give P values as exact values whenever suitable.*
☒	☐	For Bayesian analysis, information on the choice of priors and Markov chain Monte Carlo settings
☒	☐	For hierarchical and complex designs, identification of the appropriate level for tests and full reporting of outcomes
☐	☒	Estimates of effect sizes (e.g. Cohen's *d*, Pearson's *r*), indicating how they were calculated

Our web collection on statistics for biologists contains articles on many of the points above.

Software and code

Policy information about availability of computer code

Data collection	Protein colocalization studies were performed using Zeiss Zen 2.3 blue edition confocal acquisition software.
Data analysis	Quantification of protein colocalization was analyzed by Image J 1.50i sofeware. Statistical analysis was used Prism 6 or Excel.

For manuscripts utilizing custom algorithms or software that are central to the research but not yet described in published literature, software must be made available to editors and reviewers. We strongly encourage code deposition in a community repository (e.g. GitHub). See the Nature Portfolio guidelines for submitting code & software for further information.

Data

Policy information about availability of data

All manuscripts must include a data availability statement. This statement should provide the following information, where applicable:

- Accession codes, unique identifiers, or web links for publicly available datasets
- A description of any restrictions on data availability
- For clinical datasets or third party data, please ensure that the statement adheres to our policy

Source data including all raw data generated in this study are provided with this paper. The following figures have associated raw data listed in the source data file: Figure 1a-1j, 2b, 2c, 2e, 2f, 2g, 2h, 3d, 3f, 3g, 4f, 4g, 4i, 4j, 5e, 6a, 6b, 6d, 6e, 7c, 7e, 7f, 7i and Supplementary Figure 1j, 2a-2c, 2f, 2g, 3b-3d, 3f, 4b, 4f-4m, 5a-5c, 6a-6c, 6e, 7a-7d, 8a, 8c, 8d, 8e, 8g, 8h, 8i, and 8j.

Research involving human participants, their data, or biological material

Policy information about studies with human participants or human data. See also policy information about sex, gender (identity/presentation), and sexual orientation and race, ethnicity and racism.

Reporting on sex and gender	N/A
Reporting on race, ethnicity, or other socially relevant groupings	N/A
Population characteristics	N/A
Recruitment	N/A
Ethics oversight	N/A

Note that full information on the approval of the study protocol must also be provided in the manuscript.

Field-specific reporting

Please select the one below that is the best fit for your research. If you are not sure, read the appropriate sections before making your selection.

☒ Life sciences ☐ Behavioural & social sciences ☐ Ecological, evolutionary & environmental sciences

For a reference copy of the document with all sections, see nature.com/documents/nr-reporting-summary-flat.pdf

Life sciences study design

All studies must disclose on these points even when the disclosure is negative.

Sample size	Sample size was based on our previous experience in the experimental approach to obtain reliable results (PMID: 32541877).
Data exclusions	No data were excluded.
Replication	All findings were reliably reproduced at least twice and reached similar results.
Randomization	The experimental approach did not require samples to be randomized. All samples and related data analysis were handled in the same way in all experiments.
Blinding	The investigators were not blinded to group allocation during data collection and analysis, which is considered standard for biochemical analysis and microscopy experiments in this study. Each experiment was repeated by different investigators and reach similar results.

Reporting for specific materials, systems and methods

We require information from authors about some types of materials, experimental systems and methods used in many studies. Here, indicate whether each material, system or method listed is relevant to your study. If you are not sure if a list item applies to your research, read the appropriate section before selecting a response.

Materials & experimental systems

n/a	Involved in the study	
☐	☒	Antibodies
☐	☒	Eukaryotic cell lines
☒	☐	Palaeontology and archaeology
☒	☐	Animals and other organisms
☒	☐	Clinical data
☒	☐	Dual use research of concern
☒	☐	Plants

Methods

n/a	Involved in the study	
☒	☐	ChIP-seq
☒	☐	Flow cytometry
☒	☐	MRI-based neuroimaging

Antibodies

Antibodies used	The following antibodies were obtained from commercial sources: anti-Myc (Cell Signaling; 2276, immunofluorescence, 1:100/ western blot, 1:1000), anti-6×His (Santa Cruz; sc-803, western blot, 1:200), anti-PGK1 (Santa Cruz; sc-130335, immunofluorescence, 1:100/western blot, 1:1000), anti-phosphoPGK1 S203 (Signalway Antibody; SAB487P, western blot, 1:500), anti-β-actin (Santa Cruz;

sc-47778, western blot, 1:1,000), anti-EGFR (Cell Signaling; 4267, immunofluorescence, 1:100/western blot, 1:1000), anti-Rab11 (Cell Signaling; 5589, immunofluorescence, 1:100), anti-TfR (Santa Cruz; sc-65882, western blot, 1:500), anti-LAMP1 (Cell Signaling; 9901, immunofluorescence, 1:100/western blot, 1:1000), anti-LAMP2 (Santa Cruz; sc-18822, immunofluorescence, 1:100/western blot, 1:1000), anti-EEA1 (Cell Signaling; 2411, immunofluorescence, 1:100/western blot, 1:1000), anti-VAMP3 (Santa Cruz; sc-514843, western blot, 1:500), anti-Rab7 (Cell Signaling; 9367, immunofluorescence, 1:100), anti-ERK (Cell Signaling; 9107, western blot, 1:1000), anti-pERK (Cell Signaling; 4370, western blot, 1:2000), anti-PIP5K1A (GeneTex; GTX111953, western blot, 1:1000), anti-PIP5K1C (GeneTex; GTX105607, western blot, 1:1000) anti-CXCR4 (abcam; 124824, western blot, 1:1000), anti-Hrs (GeneTex; GTX101718, immunofluorescence, 1:100/western blot, 1:1000), anti-HD-PTP (Santa Cruz; sc-398711, western blot, 1:500), anti-CHMP4B (GeneTex; GTX64853, western blot, 1:1000). The following conjugated secondary antibodies were obtained from Jackson ImmunoResearch: horseradish peroxidase-conjugated donkey antibodies against mouse IgG (715-035-150, western blot, 1:10,000) and against rabbit IgG (711-035-152, western blot, 1:10,000), Cy2 donkey antibodies against mouse IgG (715-225-151, immunofluorescence, 1:200) and against rabbit IgG (711-225-152, immunofluorescence, 1:200), Cy3 goat antibody against mouse IgG (115-165-062, immunofluorescence, 1:200) and Cy3 donkey antibodies against rabbit IgG (711-165-152, immunofluorescence, 1:200).

Validation

For antibodies obtained from commercial souces listed above, validation was performed by the vendors. Validations summarized from vendor's websites (application key: WB-Western Blot IP-Immunoprecipitation IHC-Immunohistochemistry IF-Immunofluorescence F-Flow Cytometry).
anti-Myc (Cell Signaling; 2276): Species reactivity - all, Application - WB, IP, IHC, IF, F
anti-6×His (Santa Cruz; sc-803): Species reactivity - all, Application - WB, IP
anti-PGK1 (Santa Cruz; sc-130335): Species reactivity - mouse, rat and human, Application - WB, IP, IHC, IF
anti-phosphoPGK1 S203 (Signalway Antibody; SAB487P): Species reactivity - mouse, rat and human, Application - WB
anti-β-actin (Santa Cruz; sc-47778): Species reactivity - mouse, rat, human, avian, bovine, canine, porcine, rabbit, Dictyostelium discoideum and Physarum polycephalum, Application - WB, IP, IHC, IF, F
anti-EGFR (Cell Signaling; 4267): Species reactivity - mouse, monkey and human, Application - WB, IP, IHC, IF, F
anti-Rab11 (Cell Signaling; 5589): Species reactivity - mouse, rat, monkey and human, Application - WB, IP, IF
anti-TfR (Santa Cruz; sc-65882): Species reactivity - mouse, rat and human, Application - WB, IP, IHC, IF, F
anti-LAMP1 (Cell Signaling; 9091): Species reactivity - monkey and human, Application - WB, IP, IHC, IF, F
anti-LAMP2 (Santa Cruz; sc-18822): Species reactivity - human, Application - WB, IP, IHC, IF, F
anti-EEA1 (Cell Signaling; 2411): Species reactivity - mouse, rat, monkey and human, Application - WB, IP, IF
anti-VAMP3 (Santa Cruz; sc-514843): Species reactivity - mouse, rat and human, Application - WB, IP, IF
anti-Rab7 (Cell Signaling; 9367): Species reactivity - mouse, rat, monkey and human, Application - WB, IP, IF
anti-ERK (Cell Signaling; 9107): Species reactivity - mouse, rat, monkey, human, hamster, zebrafish, mink and bovine, Application - WB
anti-pERK (Cell Signaling; 4370): Species reactivity - mouse, rat, monkey, human, hamster, zebrafish, mink and bovine, Application - WB, IP, IHC, IF, F
anti-PIP5K1A (GeneTex; GTX111953): Species reactivity - human and mouse, Application - WB, IHC, IF
anti-PIP5K1C (GeneTex; GTX105607): Species reactivity - human, Application - WB
anti-CXCR4 (abcam; 124824): Species reactivity - mouse, rat and human, Application - WB, IHC, IF, F
anti-Hrs (GeneTex; GTX101718): Species reactivity - mouse, rat and human, Application - WB, IHC, IF
anti-HD-PTP (Santa Cruz; sc-398711): Species reactivity - mouse, rat and human, Application - WB, IP, IF
anti-CHMP4B (GeneTex; GTX64853): Species reactivity - mouse, rat and human, Application - WB

Eukaryotic cell lines

Policy information about cell lines and Sex and Gender in Research

Cell line source(s)	HeLa and A549 cells were obtained from ATCC.
Authentication	Cells were authenticated by ATCC via Short Tamdem Repeat (STR).
Mycoplasma contamination	Cells were documented by ATCC to be free of mycoplasma contamination.
Commonly misidentified lines (See ICLAC register)	No commonly misidentified cell lines were used in this study.
