# Supplementary material for: Phosphoglycerate kinase 1 acts as a cargo adaptor to promote EGFR transport to the lysosome
Source: Nat Commun. 2024 Feb 3;15:1021. doi: 10.1038/s41467-024-45443-4 (PMC10838266; doi:10.1038/s41467-024-45443-4)

Fig 1j

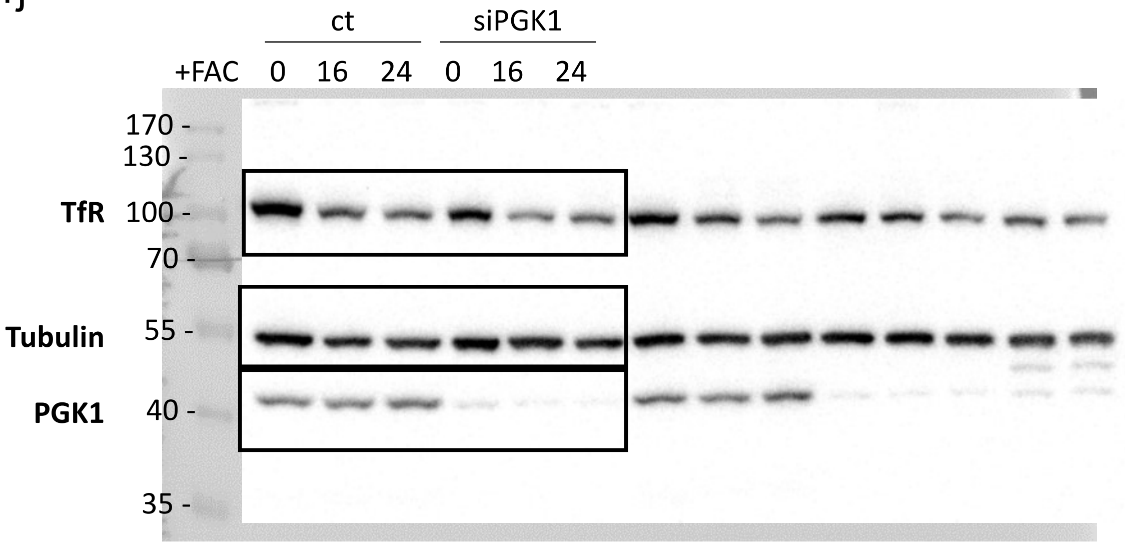
ct
siPGK1
+FAC 0 16 24 0 16 24
170 -
130 -
TfR 100 -
70 -
Tubulin 55 -
PGK1 40 -
35 -

Fig 2a

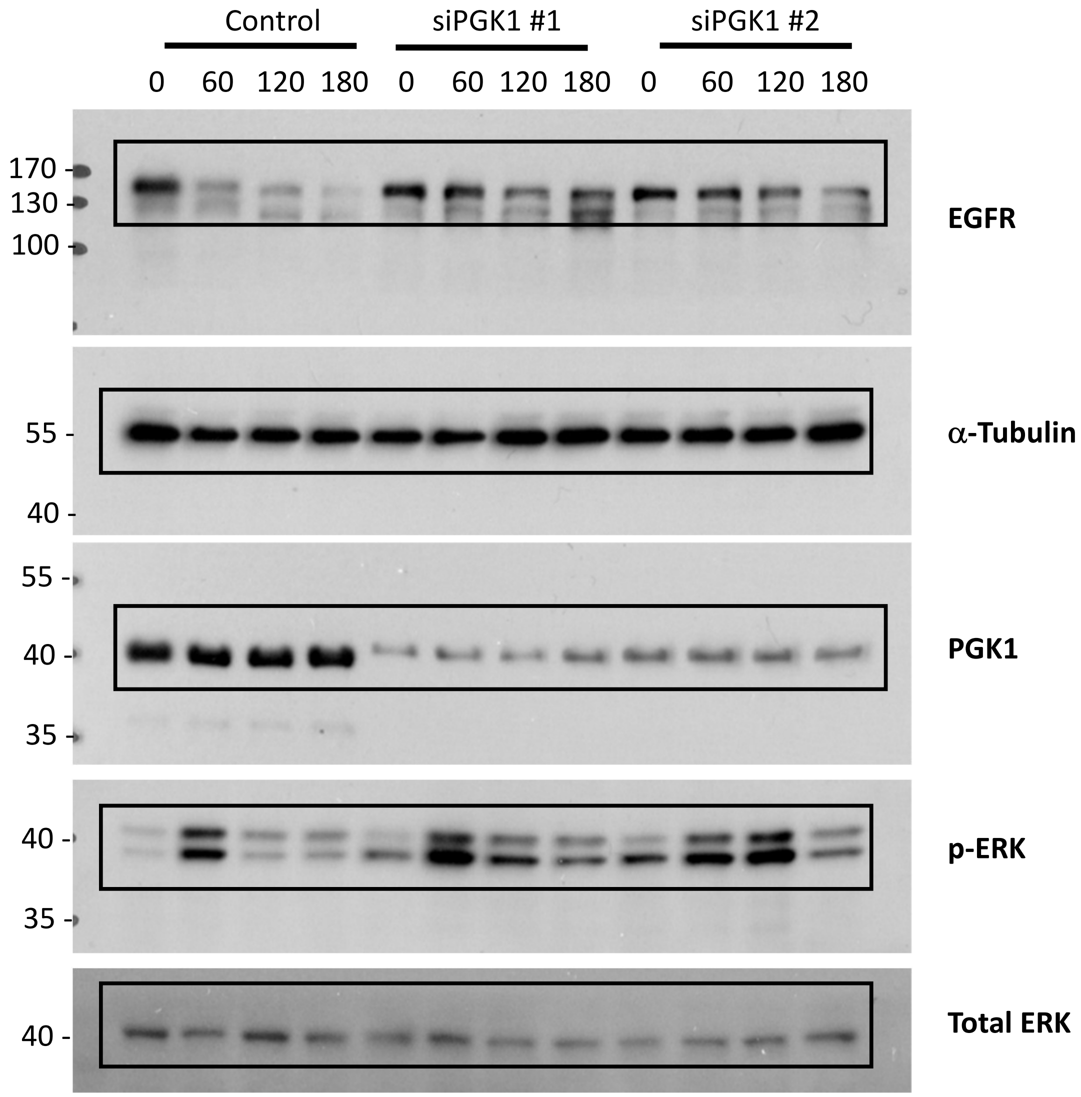
Control
siPGK1 #1
siPGK1 #2
0 60 120 180 0 60 120 180 0 60 120 180
170
130
100
EGFR
55
40
α-Tubulin
55
40
35
PGK1
40
35
p-ERK
40
Total ERK

Fig 2d

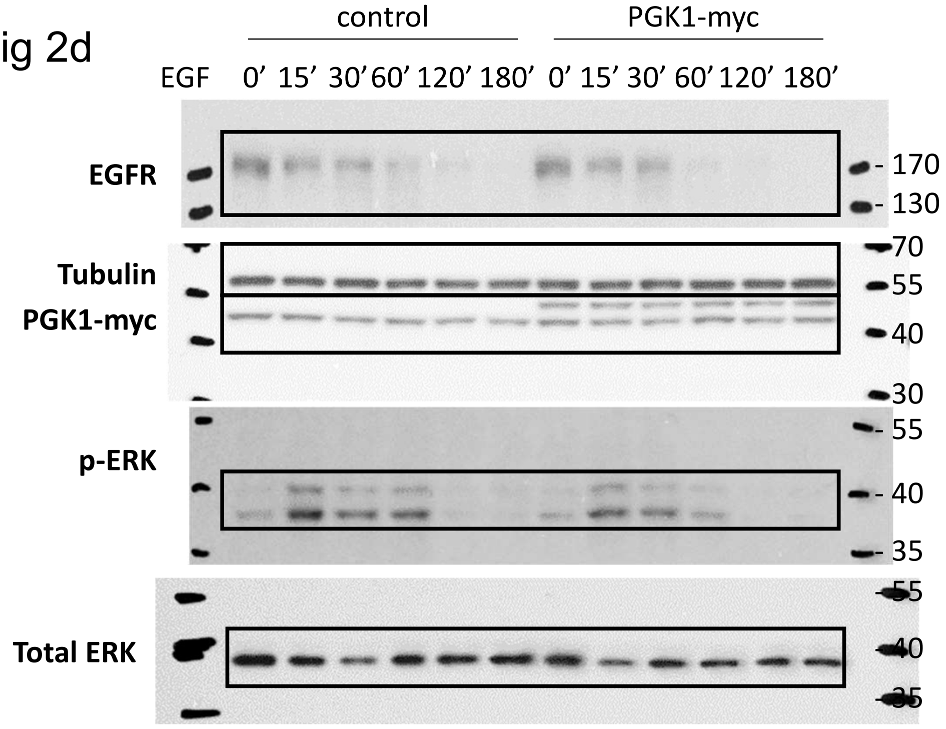
control
PGK1-myc
EGF 0' 15' 30' 60' 120' 180' 0' 15' 30' 60' 120' 180'
EGFR
170
130
Tubulin
PGK1-myc
70
55
40
30
p-ERK
55
40
35
Total ERK
55
40
35

Fig 2g

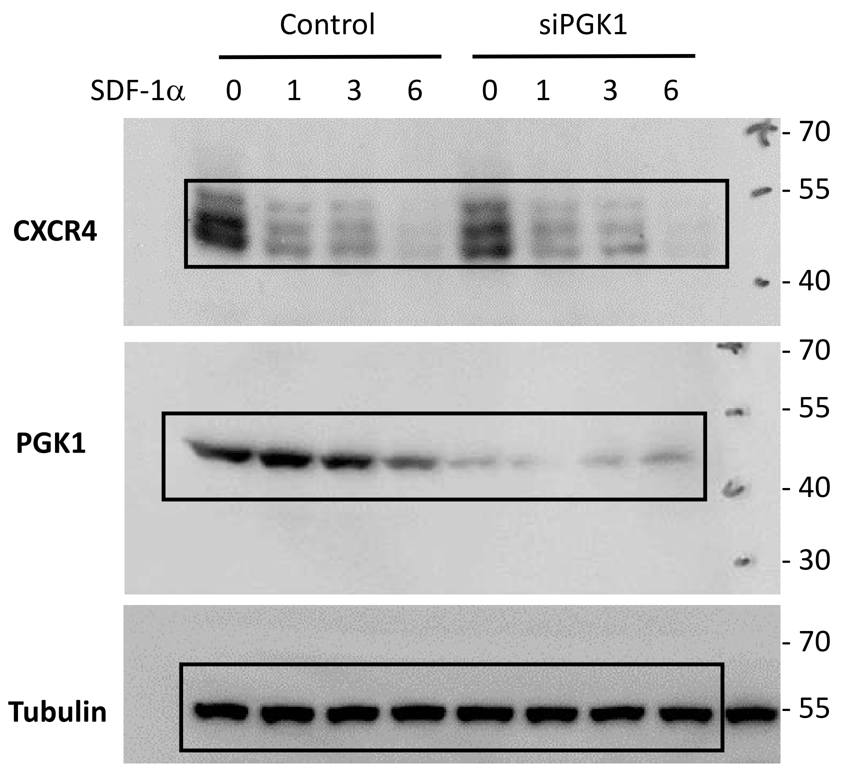
Control
siPGK1
SDF-1α 0 1 3 6 0 1 3 6
CXCR4
PGK1
Tubulin
- 70
- 55
- 40
- 70
- 55
- 40
- 30
- 70
- 55

Fig 3a

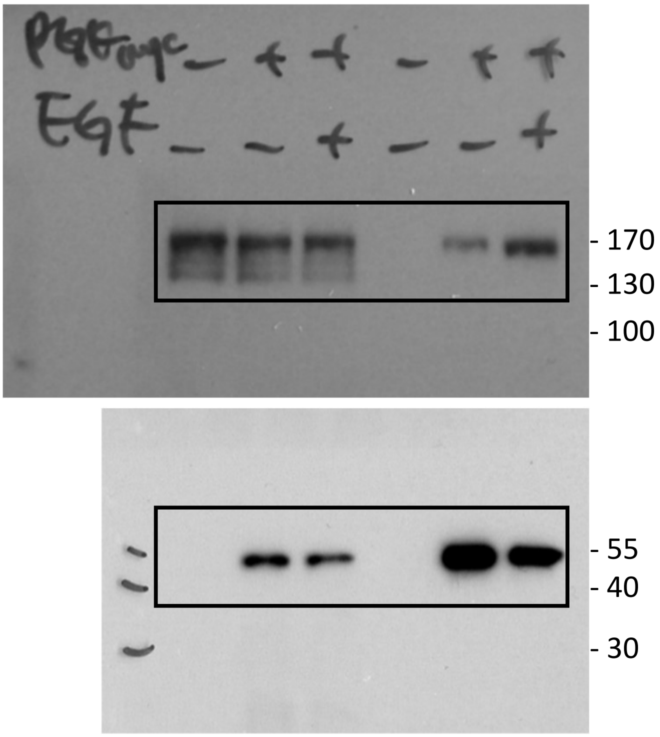

- 170
- 130
- 100
- 55
- 40
- 30

Fig 3b

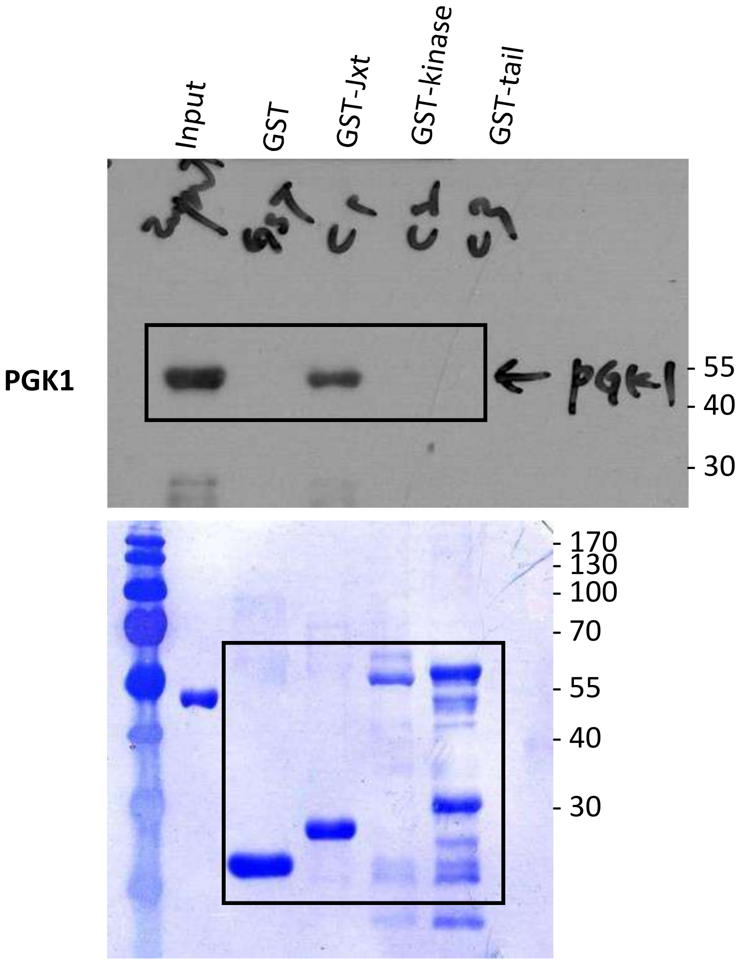

Input
GST
GST-Jxt
GST-kinase
GST-tail
PGK1
- 55
- 40
- 30
- 170
- 130
- 100
- 70
- 55
- 40
- 30

Fig 3c

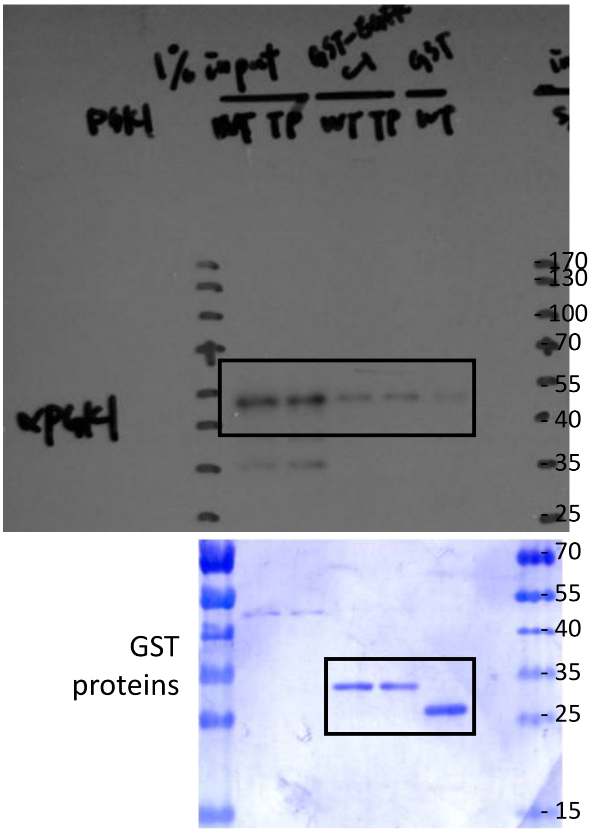
170
130
100
70
55
40
35
25
GST
proteins
70
55
40
35
25
15

Fig 3e

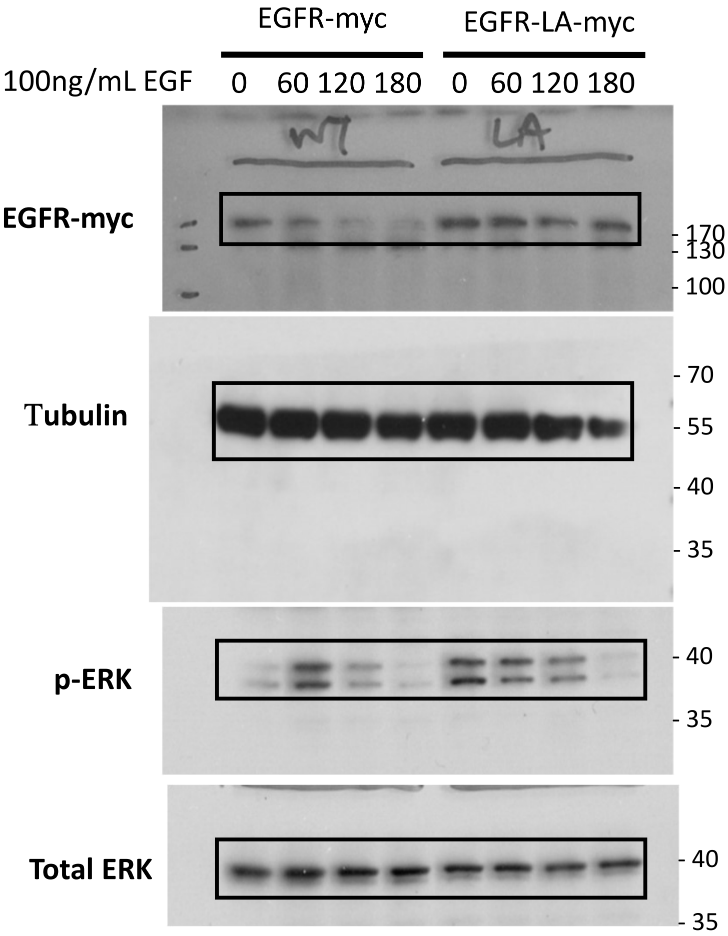
EGFR-myc
EGFR-LA-myc
100ng/mL EGF
0 60 120 180
0 60 120 180
EGFR-myc
170
130
100
Tubulin
70
55
40
35
p-ERK
40
35
Total ERK
40
35

Fig 3h

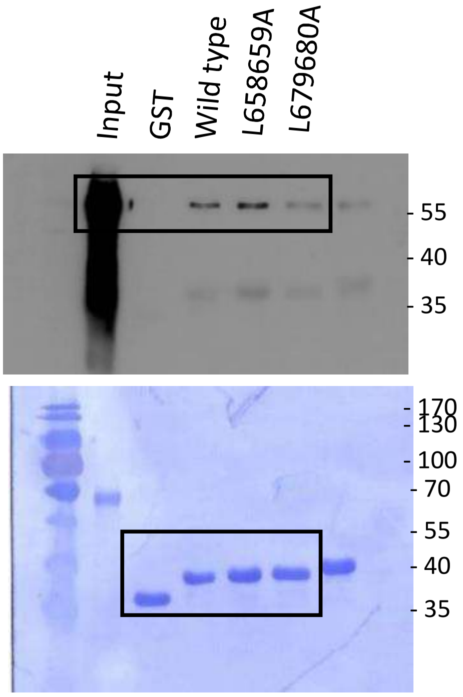
Input
GST
Wild type
L658659A
L679680A
- 55
- 40
- 35
- 170
- 130
- 100
- 70
- 55
- 40
- 35

Fig 3i

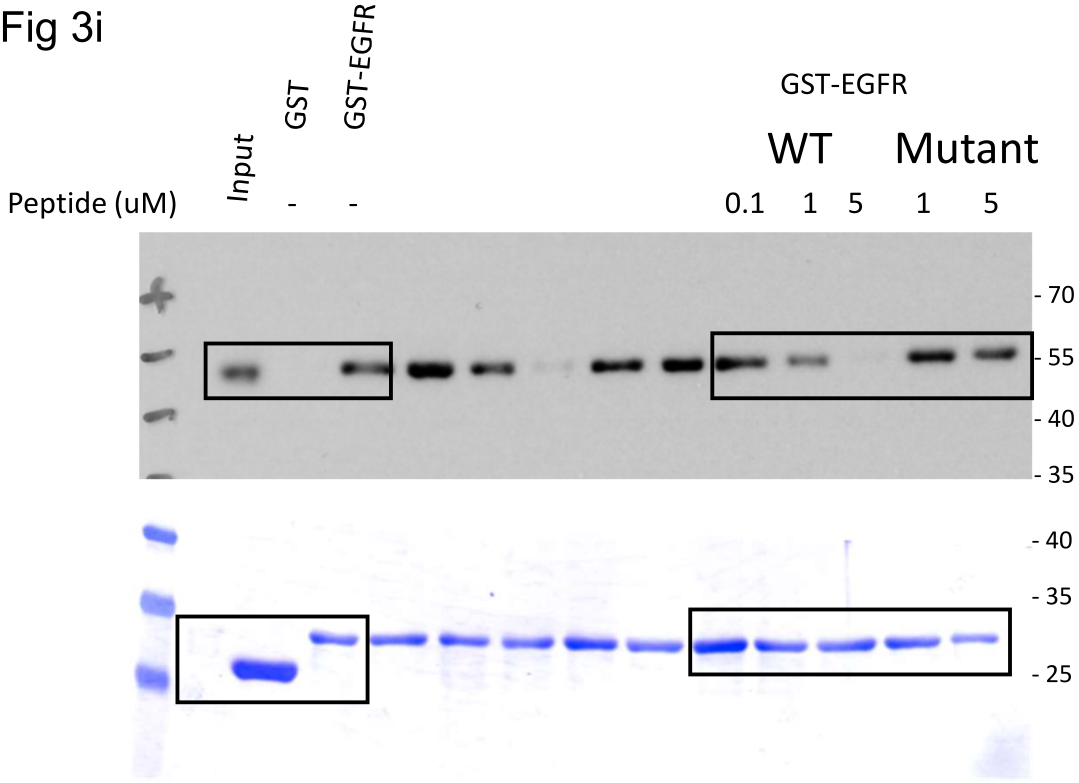
GST-EGFR
GST
GST-EGFR
Input
WT
Mutant
Peptide (uM)
-
-
0.1
1
5
1
5
- 70
- 55
- 40
- 35
- 40
- 35
- 25

Fig 3j

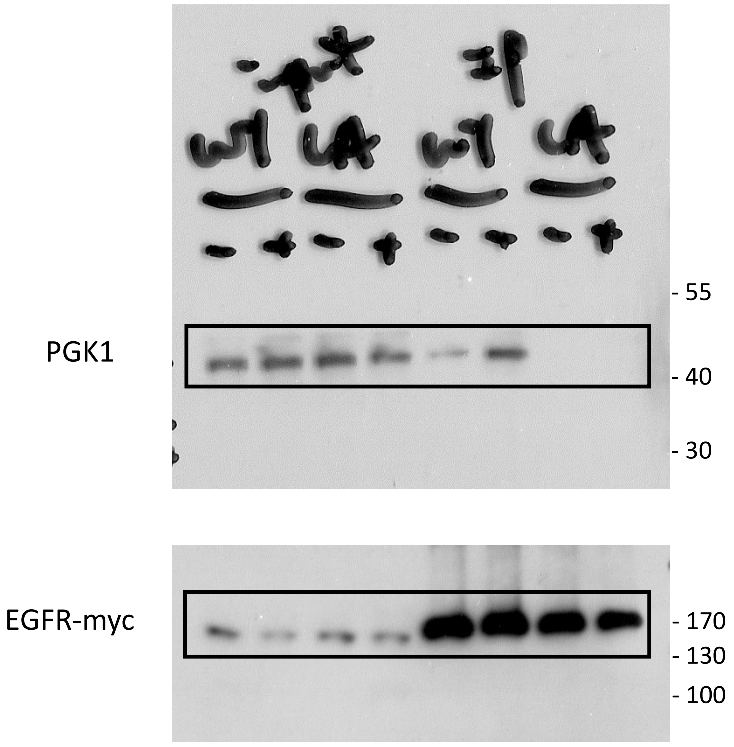

Fig 4a

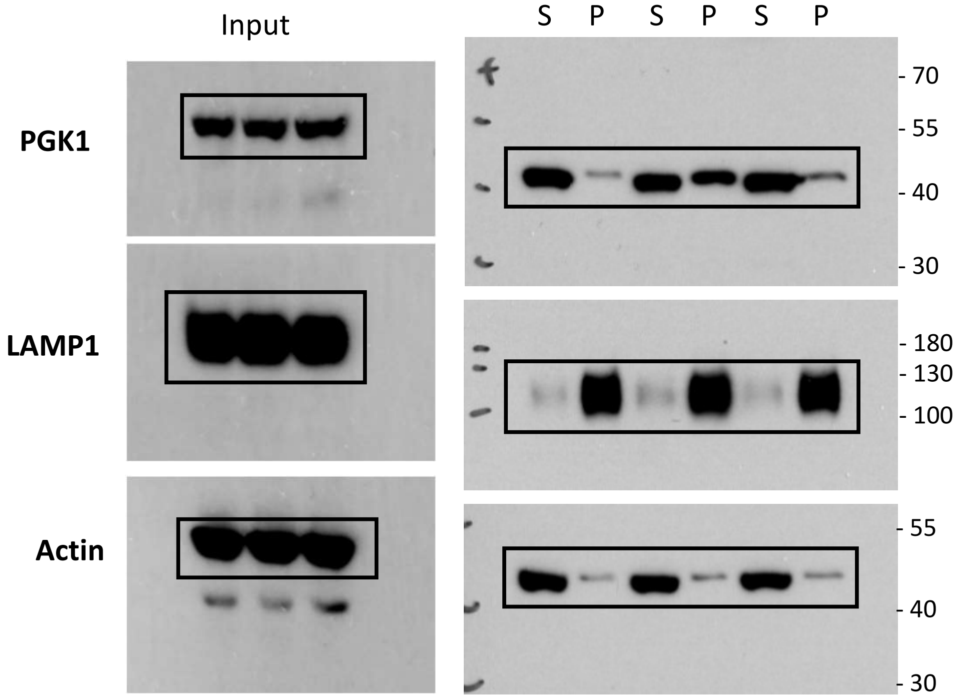
Input
S P S P S P
PGK1
LAMP1
Actin
- 70
- 55
- 40
- 30
- 180
- 130
- 100
- 55
- 40
- 30

Fig 4b

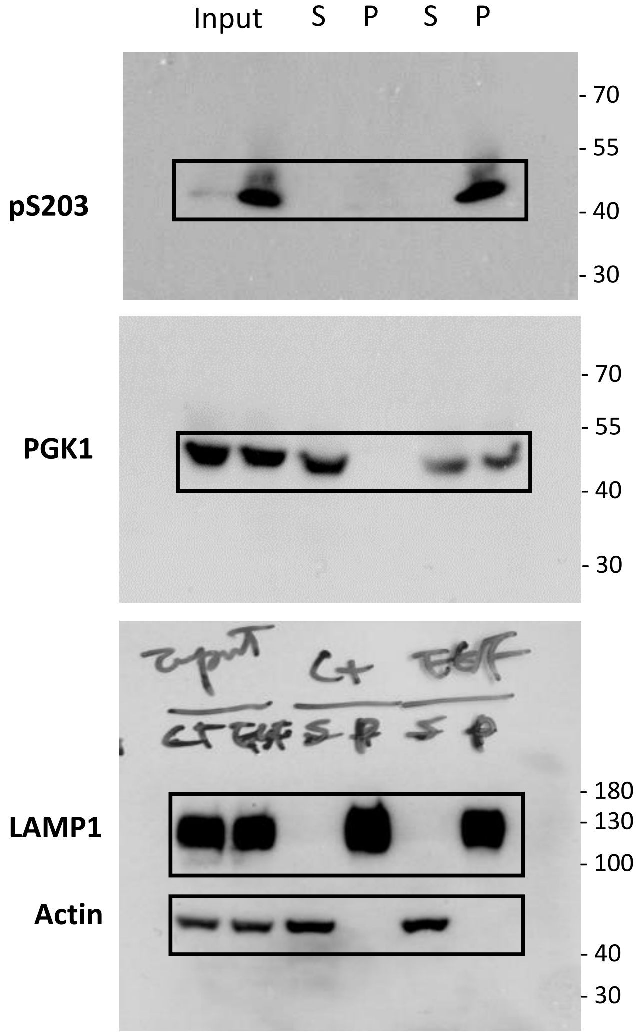
Input S P S P
pS203
PGK1
LAMP1
Actin
- 70
- 55
- 40
- 30
- 70
- 55
- 40
- 30
- 180
- 130
- 100
- 40
- 30

Fig 4c

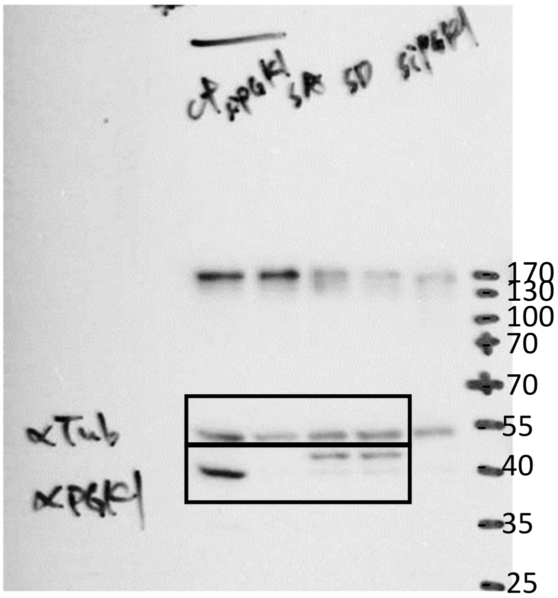

170
130
100
70
70
55
40
35
25

Fig 4d

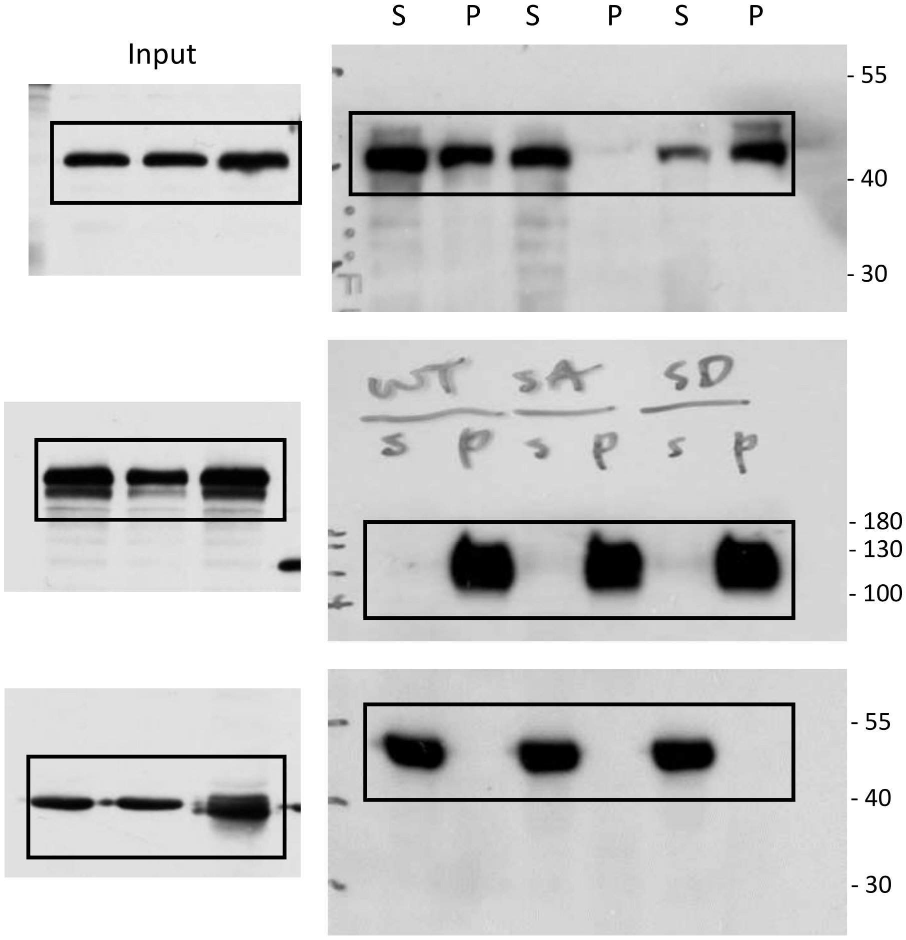

Input
S
P
S
P
S
P
- 55
- 40
- 30
WT
SA
SD
- 180
- 130
- 100
- 55
- 40
- 30

Fig 4e

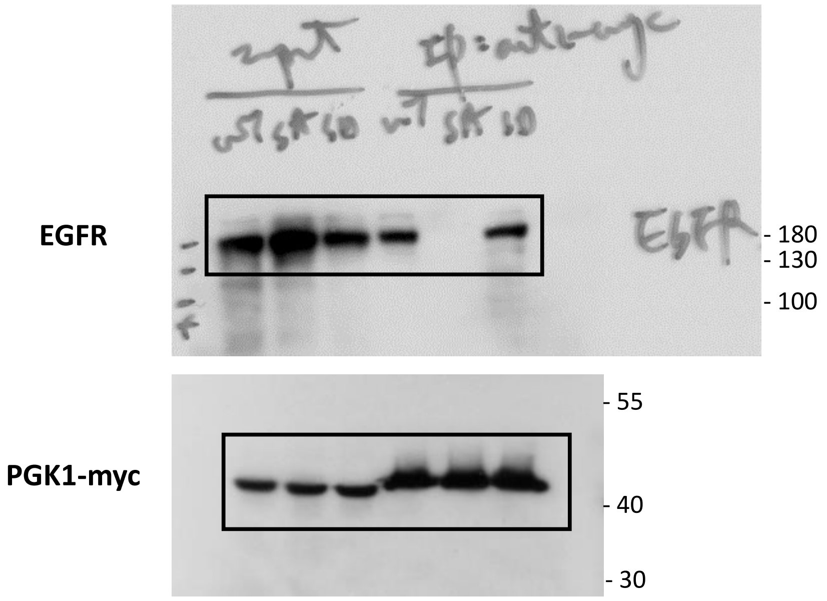

Fig 4h

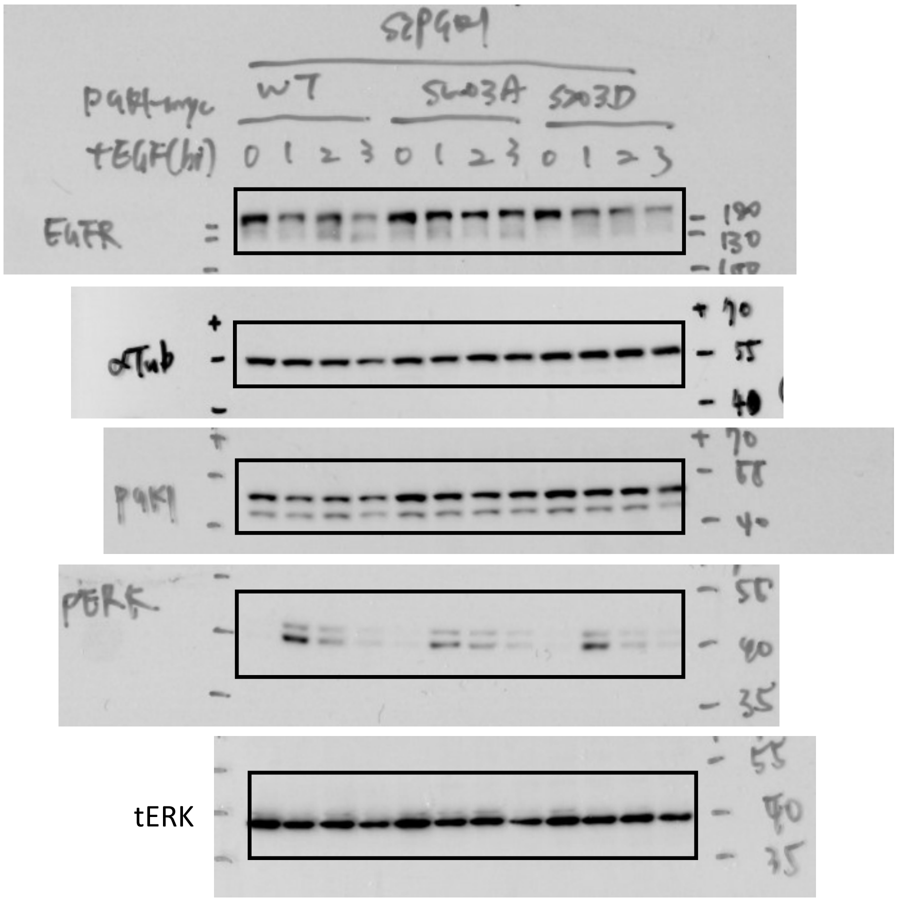

Fig 4k

PGK1

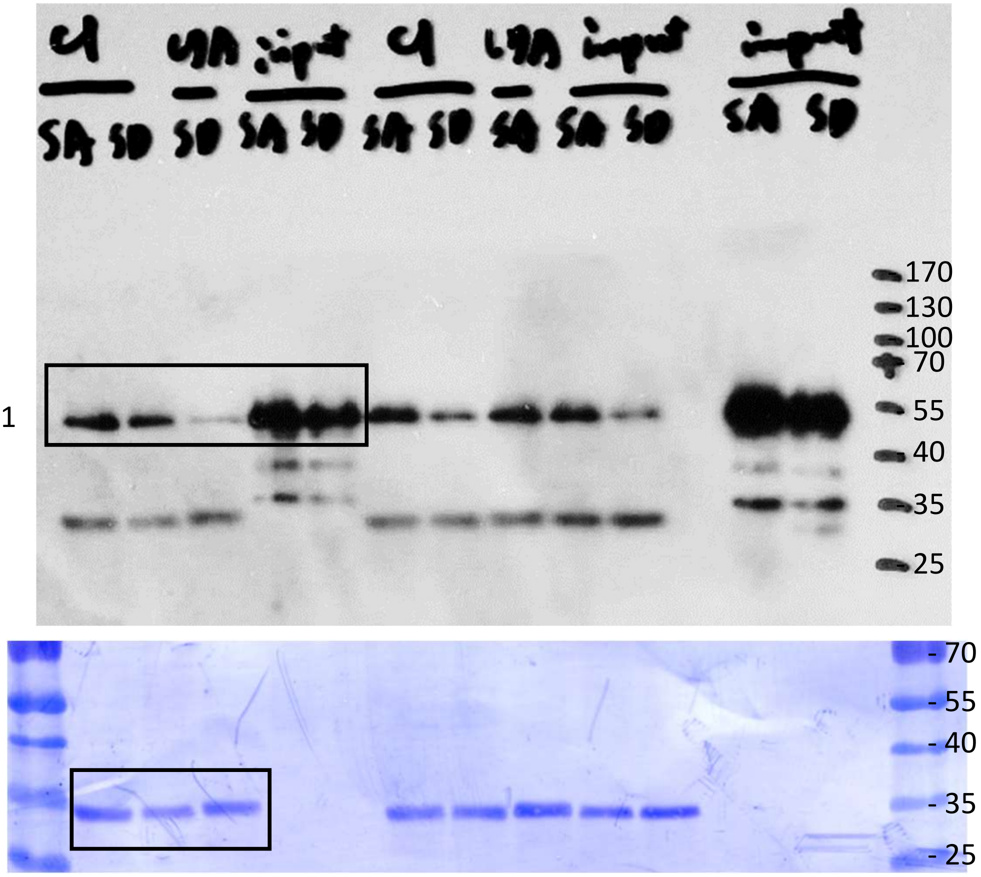

GST proteins

Fig 5a

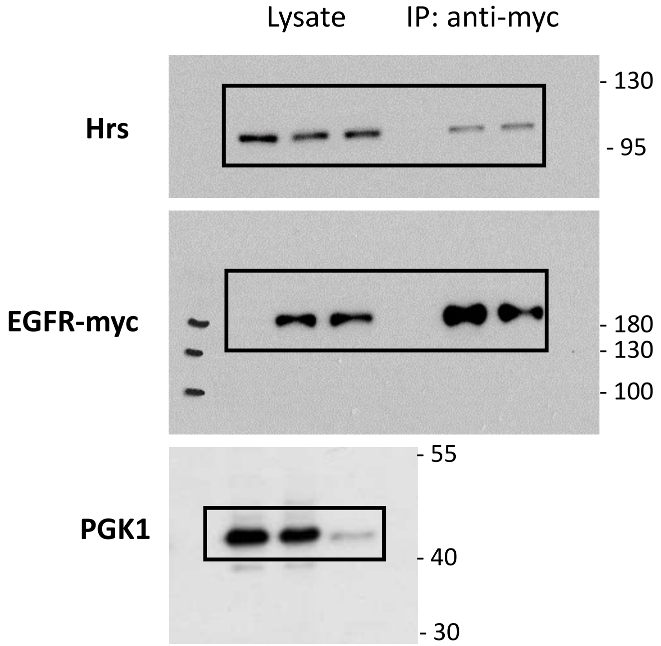

Lysate
IP: anti-myc
Hrs
EGFR-myc
PGK1
- 130
- 95
- 180
- 130
- 100
- 55
- 40
- 30

Fig 5b

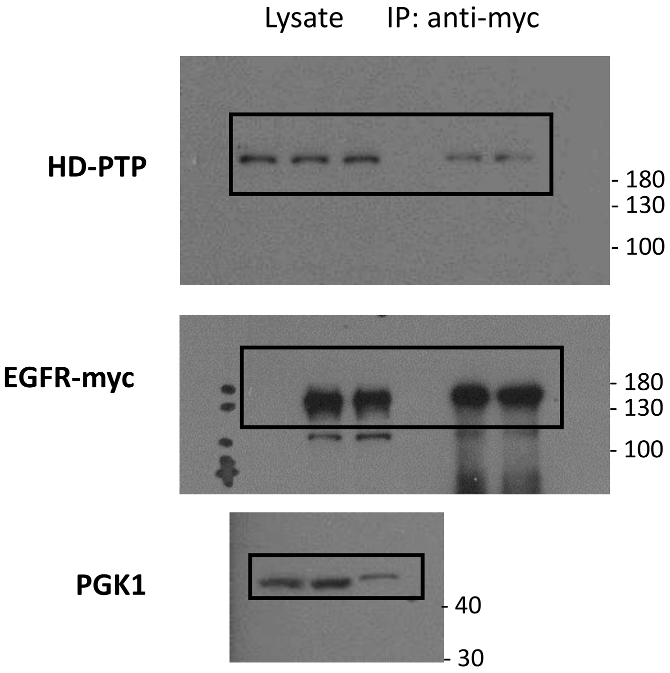

Lysate
IP: anti-myc
HD-PTP
EGFR-myc
PGK1
- 180
- 130
- 100
- 180
- 130
- 100
- 40
- 30

Fig 5c

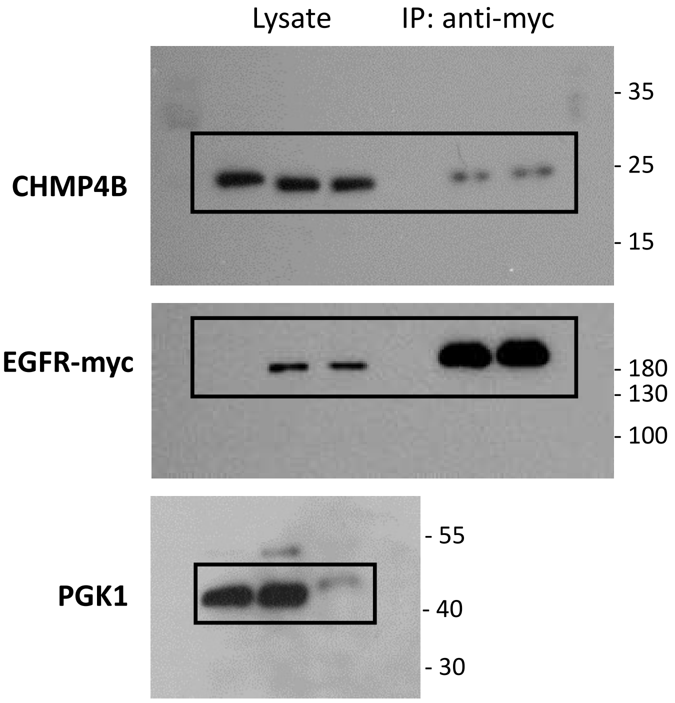
Lysate
IP: anti-myc
CHMP4B
EGFR-myc
PGK1
- 35
- 25
- 15
- 180
- 130
- 100
- 55
- 40
- 30

Fig 5d

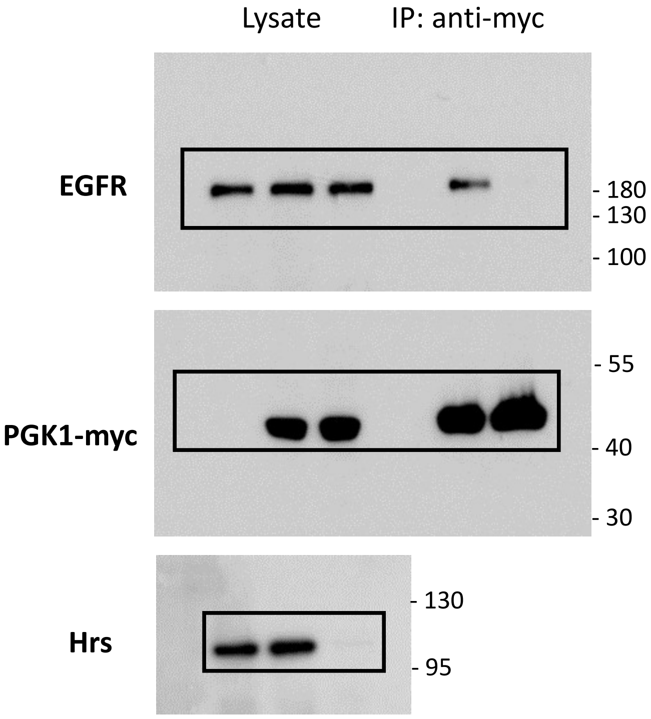
Lysate
IP: anti-myc
EGFR
PGK1-myc
Hrs
- 180
- 130
- 100
- 55
- 40
- 30
- 130
- 95

Fig 6a

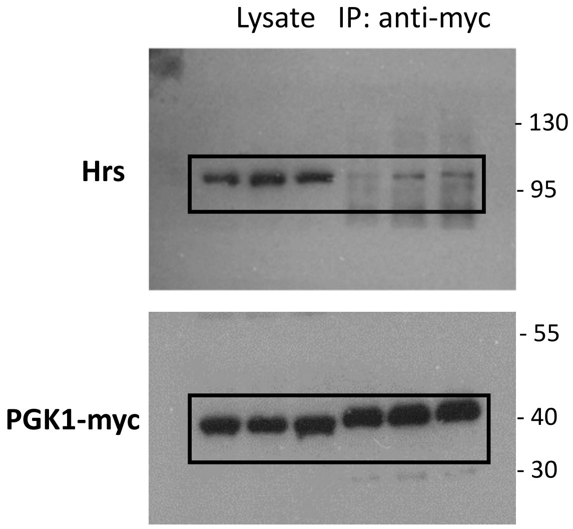

Lysate IP: anti-myc
Hrs
PGK1-myc
- 130
- 95
- 55
- 40
- 30

Fig 6c

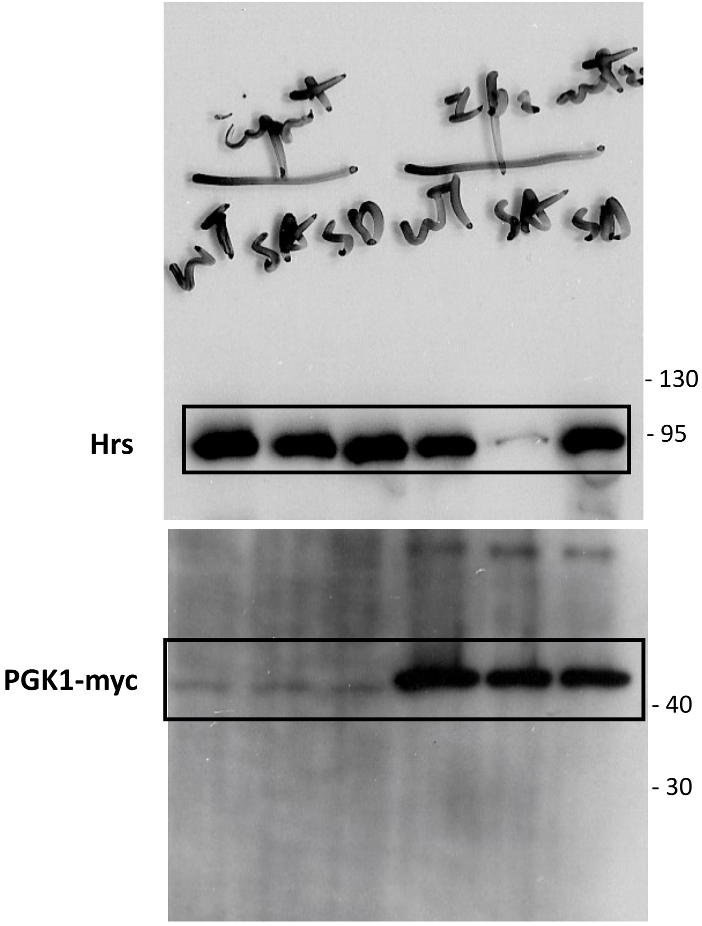

Hrs
PGK1-myc
- 130
- 95
- 40
- 30

Fig 6f

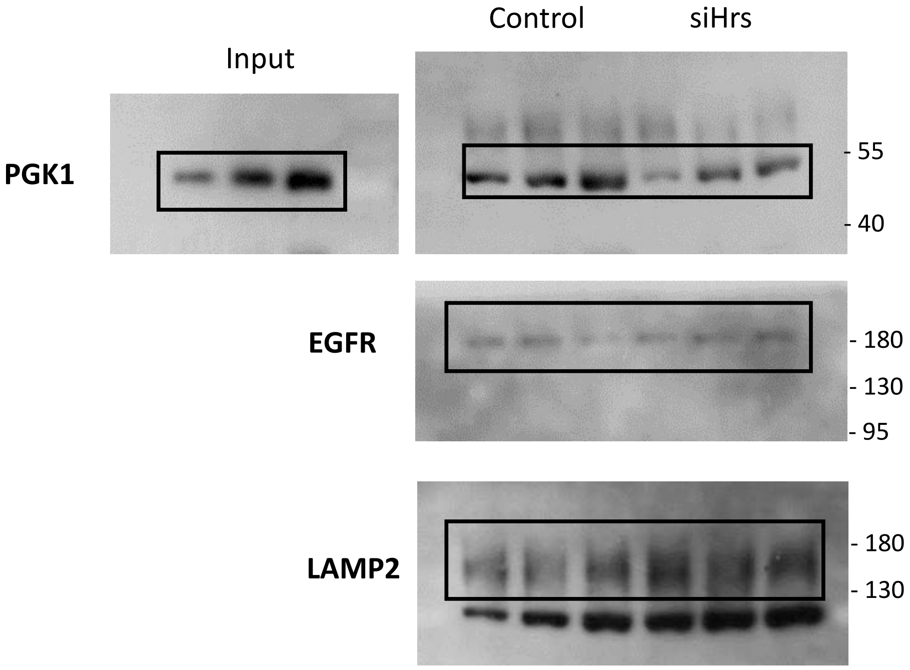

Fig 7a

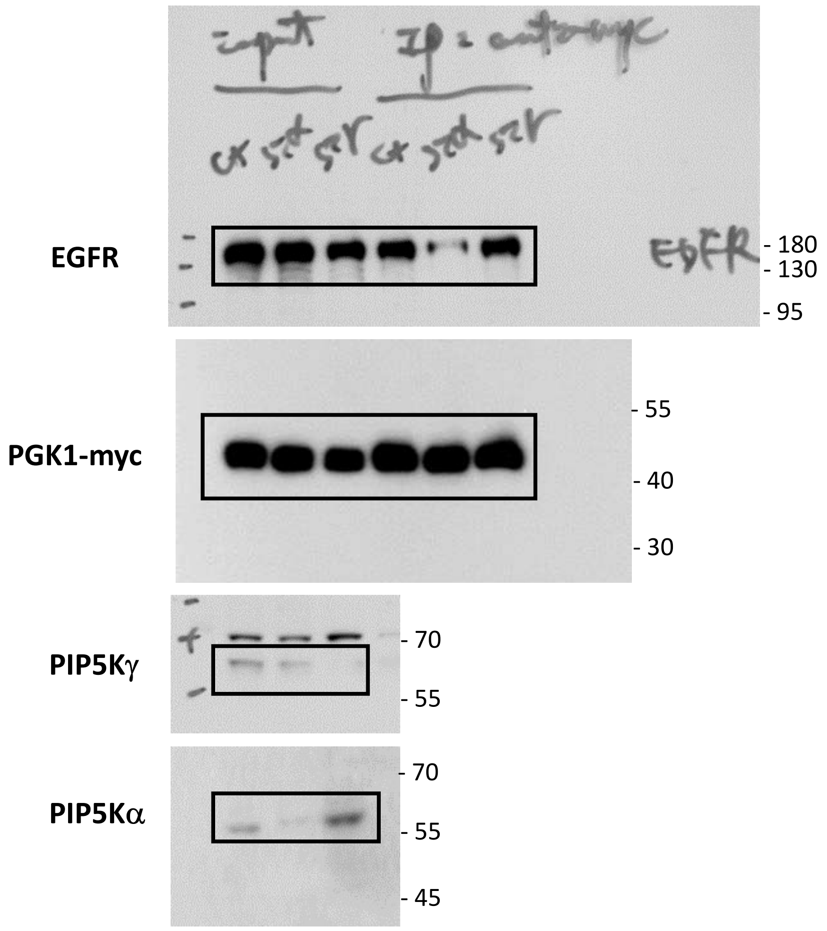
EGFR
- 180
- 130
- 95
PGK1-myc
- 55
- 40
- 30
PIP5Kγ
- 70
- 55
PIP5Kα
- 70
- 55
- 45

Fig 7b

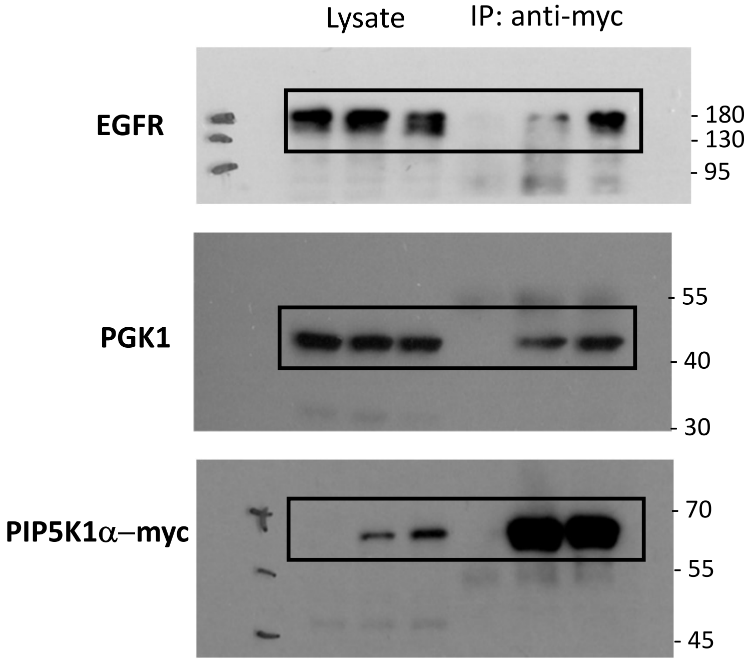
Lysate
IP: anti-myc
EGFR
- 180
- 130
- 95
PGK1
- 55
- 40
- 30
PIP5K1α–myc
- 70
- 55
- 45

Fig 7d

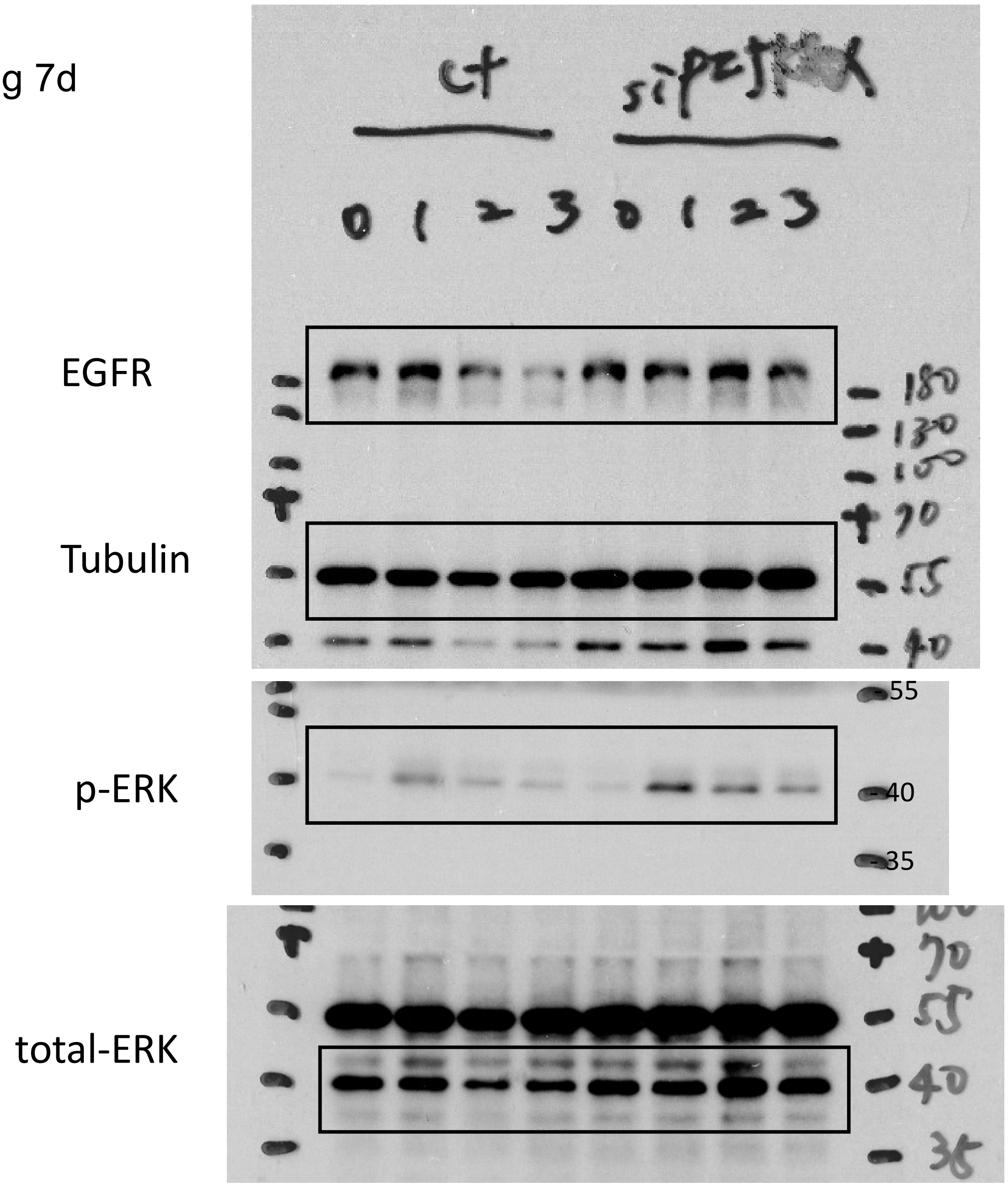
EGFR
Tubulin
p-ERK
total-ERK
55
40
35

Fig 7g

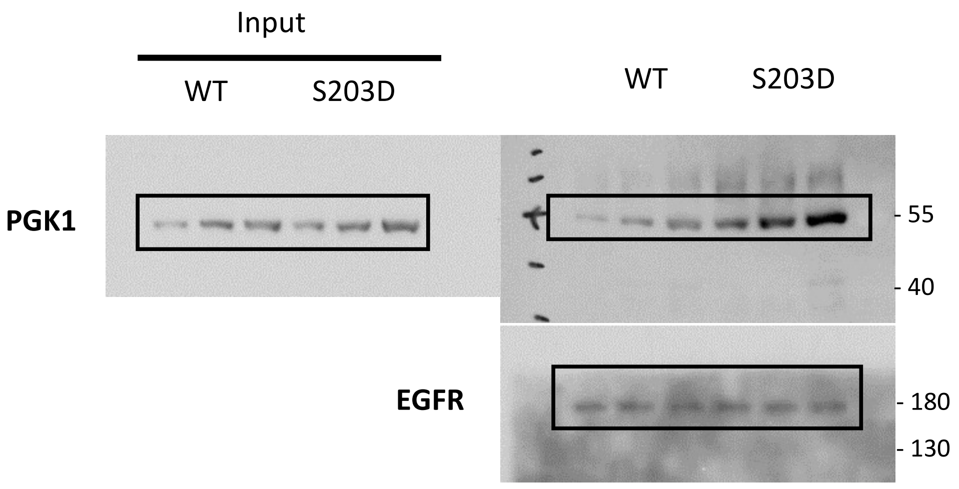
Input
WT
S203D
WT
S203D
PGK1
EGFR
- 55
- 40
- 180
- 130

Fig 7h

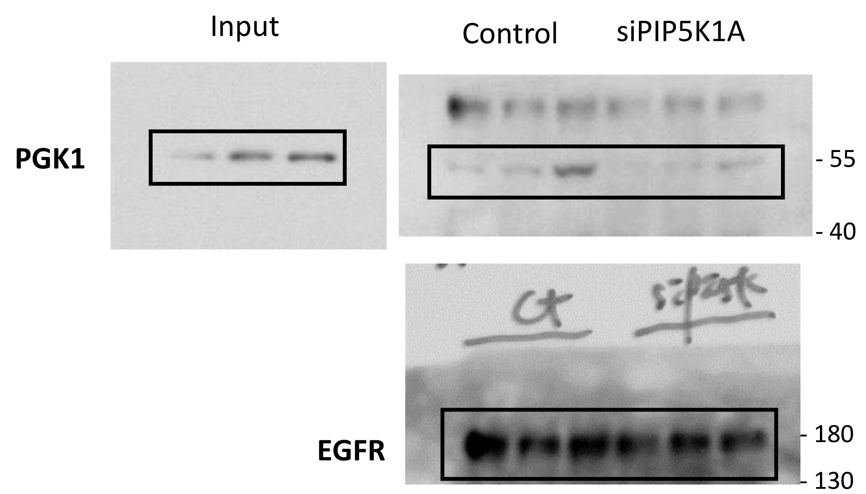
Input
Control
siPIP5K1A
PGK1
- 55
- 40
EGFR
- 180
- 130

Fig 7i

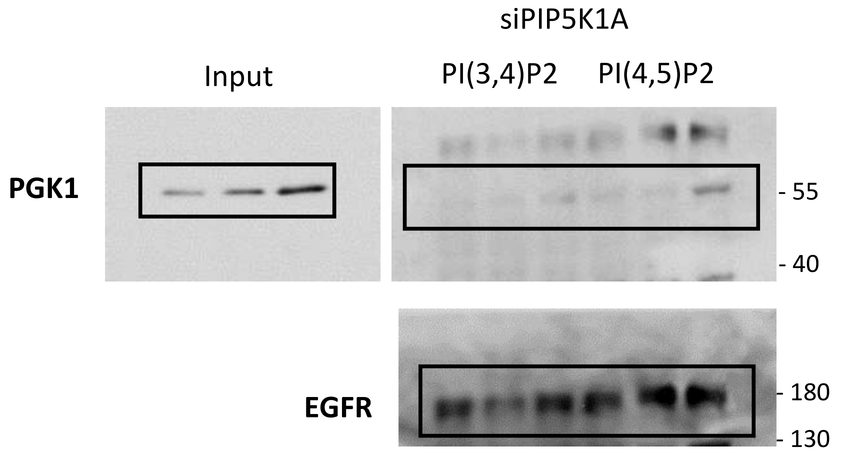
siPIP5K1A
Input
PI(3,4)P2
PI(4,5)P2
PGK1
- 55
- 40
EGFR
- 180
- 130

Supplementary Fig 2d

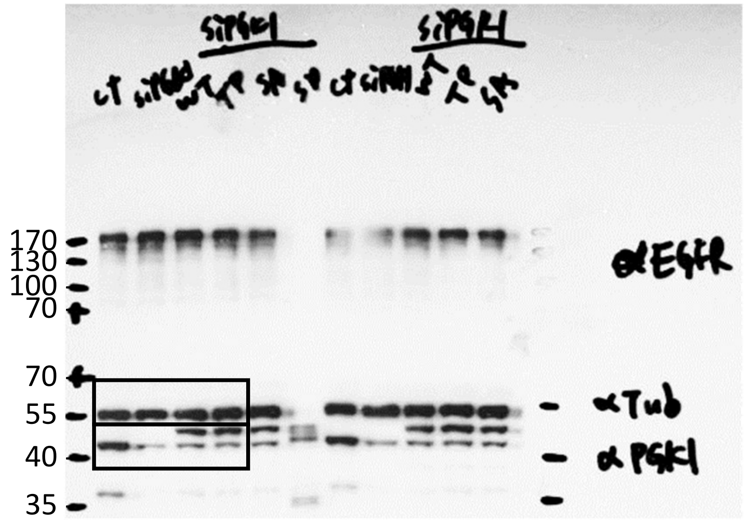

Supplementary Fig 2e

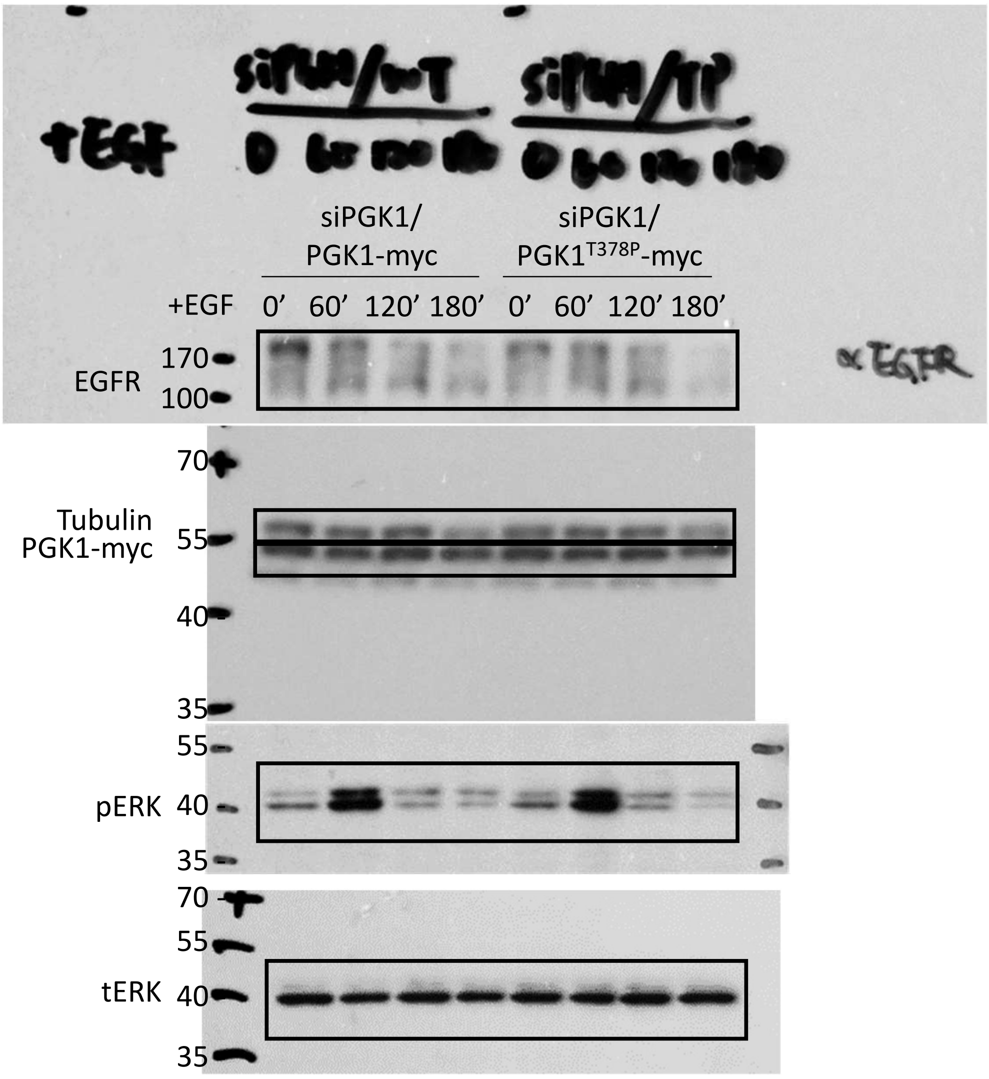

Supplementary Fig 3c

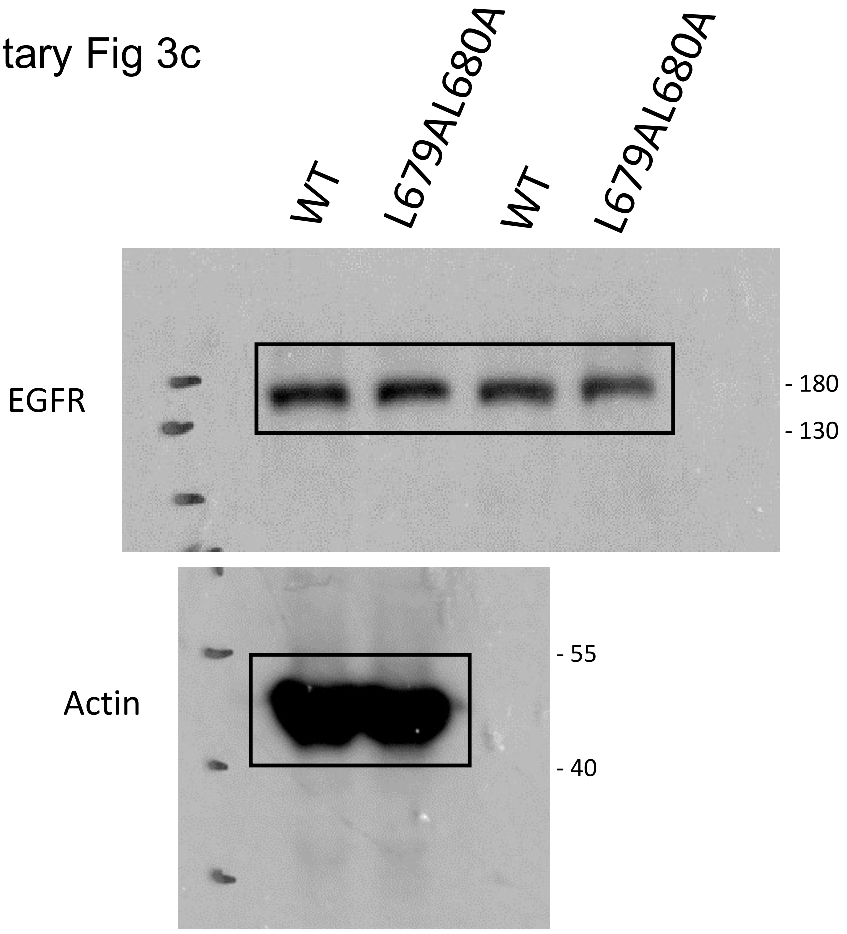

Supplementary Fig 3e

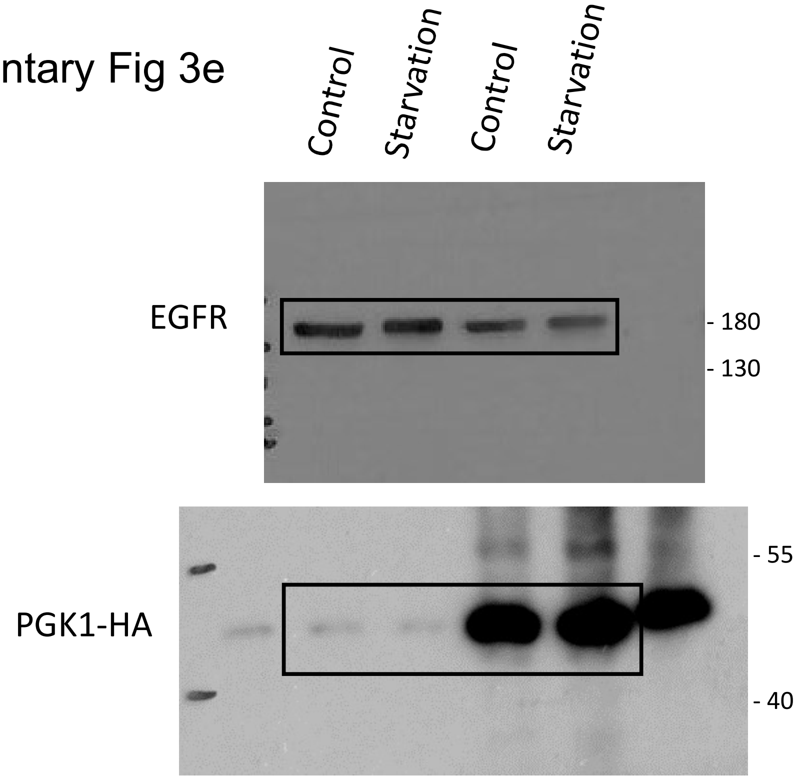

Supplementary Fig 4a

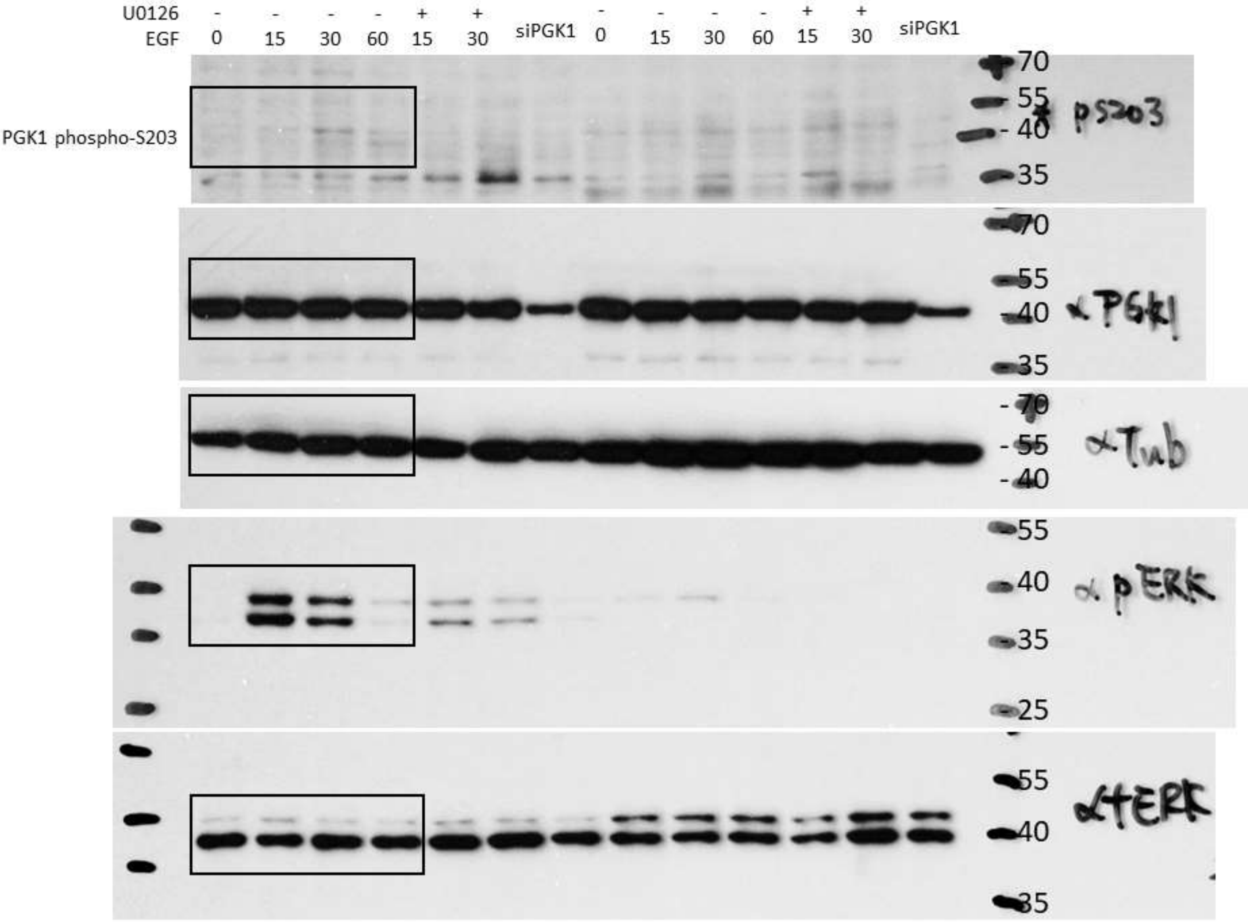

Supplementary Fig 6d

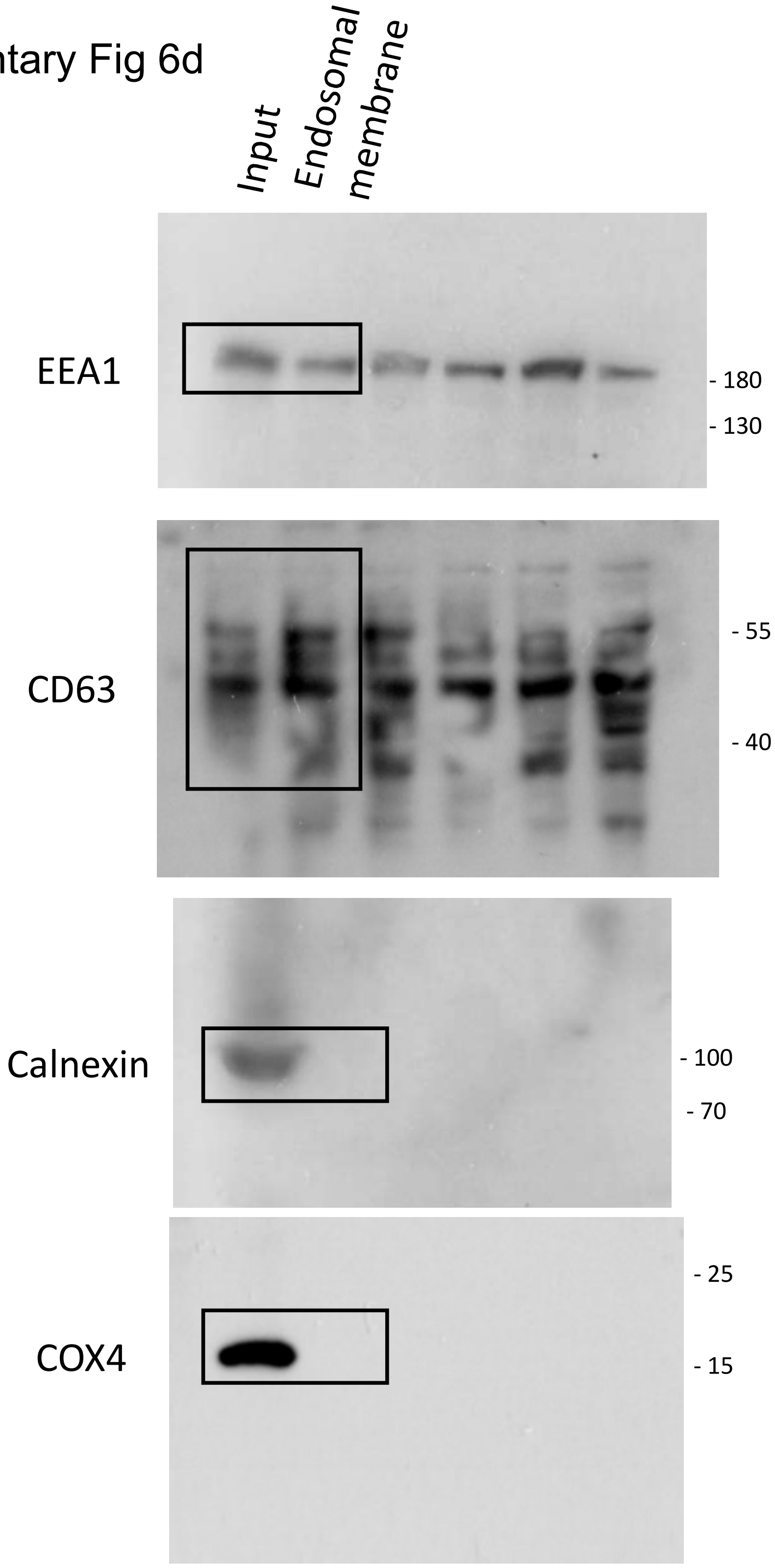

Supplementary Fig 8b

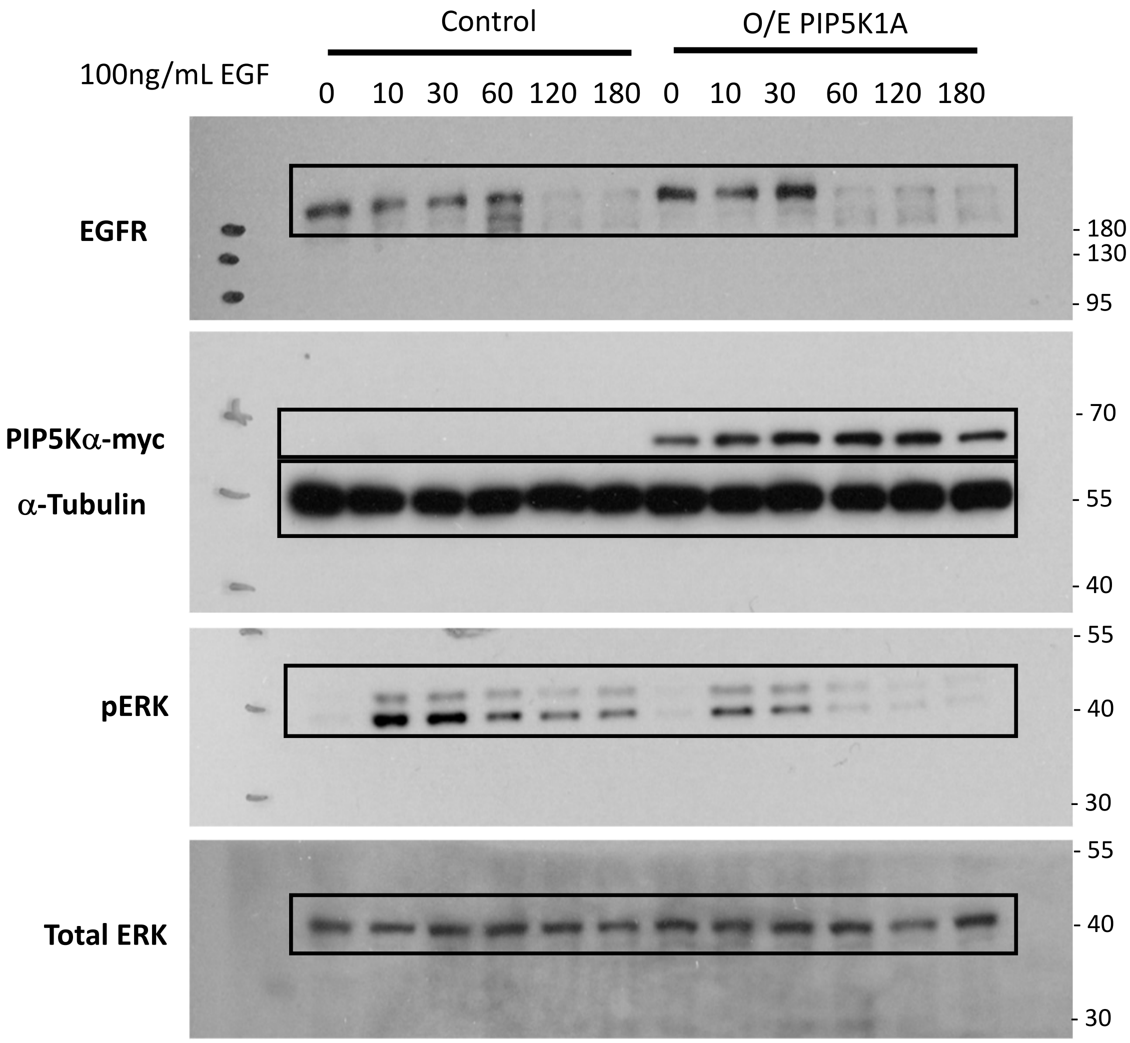

Supplementary Fig 8f

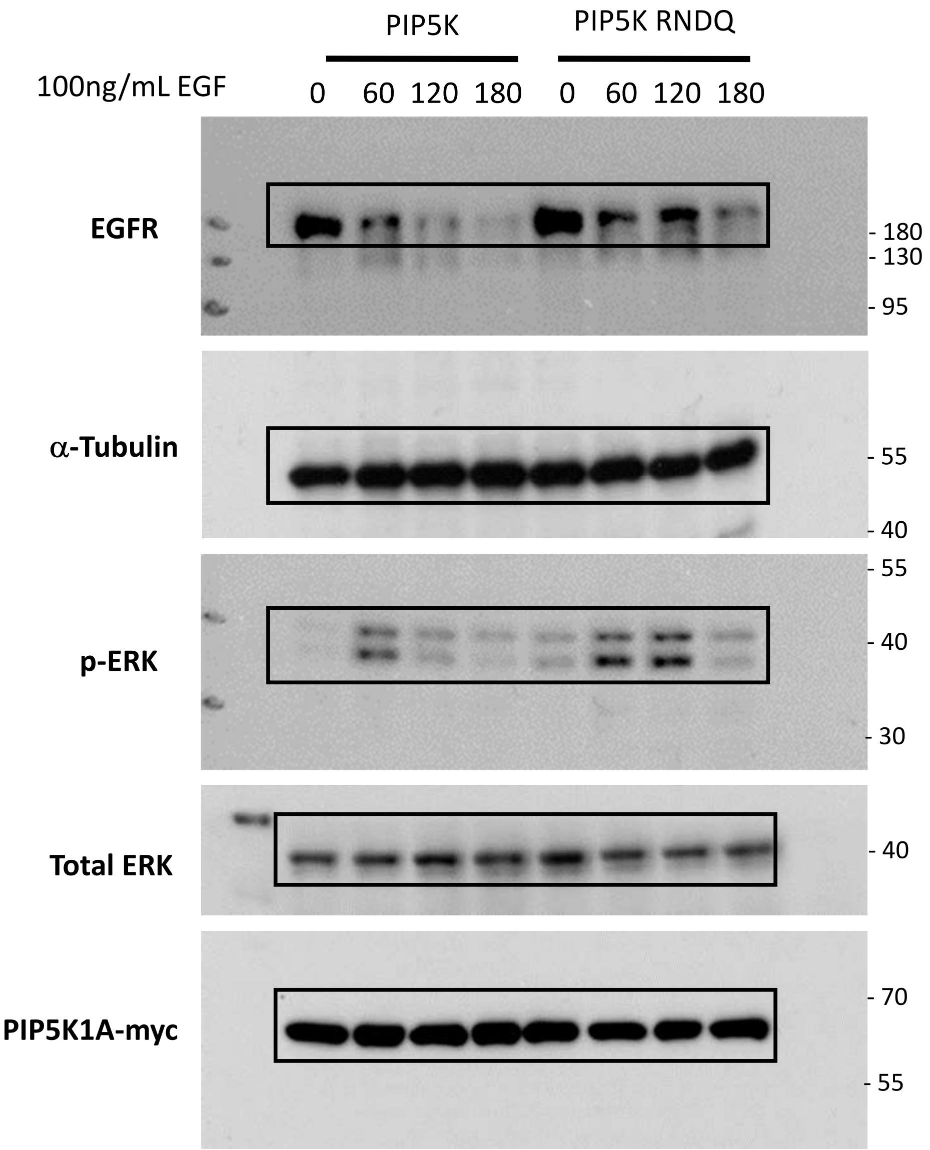

Supplement: Supplementary file 4 — Source Data [file 41467_2024_45443_MOESM4_ESM.zip › Uncropped Gel Images.pdf]
